# Methyl CpG binding protein MBD2 has a regulatory role on the BRCA1 gene expression and its modulation by resveratrol in ER+, PR+ & triple-negative breast cancer cells

**DOI:** 10.1186/s12885-024-12274-x

**Published:** 2024-05-06

**Authors:** Ram Krishna Sahu, Simran Tandon, Shalini Singh, Bhudev Chandra Das, Suresh T Hedau

**Affiliations:** 1 Division of Molecular Oncology, ICMR-National Institute of Cancer Prevention and Research, I -7, Sector – 39, Noida, Uttar Pradesh 201301 India; 2 Amity Institute of Molecular Medicine & Stem Cell Research, Amity University, Noida, Uttar Pradesh 201313 India; 3 Amity University, Mohali, Punjab 140306 India; 4 Division of Clinical Oncology, ICMR-National Institute of Cancer Prevention and Research, I -7, Sector – 39, Noida, Uttar Pradesh 201301 India

**Keywords:** Breast cancer, *BRCA1*, Methy-CpG binding protein, Epigenetics, Resveratrol

## Abstract

**Background:**

Resveratrol has demonstrated its ability to regulate *BRCA1* gene expression in breast cancer cells, and previous studies have established the binding of MBD proteins to *BRCA1* gene promoter regions. However, the molecular mechanism underlying these interactions remains to be elucidated. The aimed to evaluate the impact of MBD proteins on the regulation of *BRCA1, BRCA2*, and *p16* genes and their consequential effects on breast cancer cells.

**Methods:**

Efficacy of resveratrol was assessed using the MTT assay. Binding interactions were investigated through EMSA, ChIP, & MeIP assay. Expression analyses of MBD genes and proteins were conducted using qRT-PCR and western blotting, respectively. Functional assays, including clonogenic, migratory, and sphere formation assays were used to assess cancer cells’ colony-forming, metastatic, and tumor-forming abilities. The cytotoxicity of resveratrol on cancer cells was also tested using an apoptosis assay.

**Results:**

The study determined an IC50 of 30µM for resveratrol. MBD proteins were found to bind to the *BRCA1* gene promoter. Resveratrol exhibited regulatory effects on MBD gene expression, subsequently impacting *BRCA1* gene expression and protein levels. Higher concentrations of resveratrol resulted in reduced colony and sphere formation, decreases migration of cancer cells, and an increases number of apoptotic cells in breast cancer cells.

Impact

Identification of *MBD2-BRCA1* axis indicates their significant role in the induction of apoptosis and reduction of metastasis and proliferation in breast cancer cells. Further therapy can be designed to target these MBD proteins and resveratrol could be used along with other anticancer drugs to target breast cancer.

**Conclusions:**

In conclusion *MBD2* protein interact to the *BRCA1* gene promoter, and resveratrol modulates *MBD2* gene expression, which in turn regulates *BRCA1* gene expression, and inhibits cell proliferation, migration, and induces apoptosis in ER+, PR+ & Triple negative breast cancer cells.

**Supplementary Information:**

The online version contains supplementary material available at 10.1186/s12885-024-12274-x.

## Introduction

Breast cancer is the most commonly diagnosed cancer and the leading cause of cancer death in females worldwide. According to Globocon 2020, there will be an estimated 19.3 million new cancer cases and 10.0 million cancer-related deaths in 2020. Additionally, there will be 2.3 million (11.7%) new cases and 685,000 deaths in females worldwide due to breast cancer [1, 2]. Epigenetics plays a significant regulatory role in genes expression. DNA methylation is a well-studied epigenetic alteration that has been linked to normal mammalian development and cancer [3–5]. To elucidate the mechanism of DNA methylation, a major approach is to study proteins that directly interact with methylated DNA. There are two families of protein, termed DNA methyltransferases and methyl-CpG-binding domain (MBDs) proteins, which have been associated with DNA methylation. The methyl-binding protein family plays a significant role in the regulation of gene expression and its interpretation. The first methyl-binding protein, termed *MeCP2*, was discovered in 1989 [6]. *MeCP2* has a methyl-CpG-binding domain (MBD), which is the conserved domain of all MBD-containing proteins [7]. *STEDB1, STEDB2, BAZ2A, BAZ2B, MBD1, MBD2, MBD3, MBD4, MBD5, MBD6*, and *MeCP2* are among the 11 MBD proteins currently identified. It was observed that the secondary structure of MBD is the same among all MBD proteins, but their sequence variations contribute to the differences in their properties and functions [8]. The MBD superfamily may be split into three categories based on sequence homology: histone methyltransferases, histone acetyltransferases, and *MeCP2* MBD, which encompasses *MBD1-6* and *MeCP2* respectively. *MBD2* Mediates transcriptional repression associated with hypermethylated GSTP1 CpG islands in MCF-7 breast cancer cells [9].

Breast cancer susceptibility genes *BRCA1* and *BRCA2* encode multifunctional proteins. *BRCA1* is a human tumor suppressor gene found in all humans [10]. *BRCA2* is primarily associated with breast cancer and less frequently with ovarian cancer [11]. The *BRCA1* and *BRCA2* genes are key breast cancer risk factors, and they are involved in DNA damage repair, cell cycle control, apoptosis, and gene transcriptional regulation [12]. The *BRCA1* gene is generally expressed in breast and other tissue cells, where it repairs broken DNA or destroys cells if DNA repair isn’t possible [13]. The tumor suppressor *p16* has become more important in cancer research. The discovery of frequent *p16* gene mutations and deletions in human cancer cell lines revealed that *p16* plays a key role in carcinogenesis [14]. According to the mechanism of action, CDKI attaches to and inactivates the cyclin D-cyclin-dependent kinase 4 (or 6) complex, rendering the retinoblastoma protein inactive. As a result, transcription of essential cell-cycle regulatory proteins is suppressed, resulting in cell-cycle arrest [15, 16].

Resveratrol is a phytochemical found in grapes, berries, and peanut that has cancer-fighting properties by blocking multiple cellular pathways involved in tumor genesis, development, and promotion [17, 18]. Resveratrol is a phytoestrogen that binds to estrogen receptors and activates them, controlling the transcription of estrogen-responsive target genes including *BRCA1* and *BRCA2*, which are connected to breast cancer risk [17]. Resveratrol is a naturally occurring chemical that has been extensively researched for its potential to prevent and treat a variety of disorders, including cancer [19]. Resveratrol has been demonstrated to have various anti-cancer properties in vitro, guarding against both tumor start and cancer progression pathways [19, 20]. Resveratrol can cause tumor cells to enter apoptosis by promoting cell cycle arrest, preventing tumor-derived nitric oxide synthase production, and acting as an antioxidant to prevent DNA damage that can contribute to tumor development [20, 21].

Although it has been shown that Resveratrol regulate *BRCA1* gene expression in breast cancer cells and that MBD proteins bind to the *BRCA1* gene promoter regions, the molecular link or mechanism has yet to be established [17, 22]. We discovered the involvement of MBD protein in the control of *BRCA1, BRCA2*, and *p16*, as well as their impacts on breast cancer cells, in this work.

## Materials and methods

### Cell culture and maintenance

Cell lines provide an excellent and continuous source of tumor material for validation and genetic characterization. MCF-7, MDA-MB-231, and T-47-D breast cancer cell lines were obtained from NCCS Pune and kept in Dulbecco’s modified Eagle’s medium (Invitrogen, USA) and RPMI-1640 (Sigma, USA) supplemented with 15mM HEPES (pH 7.3), sodium bicarbonate, 10% heat-inactivated foetal bovine serum (FBS), and 1% antibiotic cocktail at 37°C in a humidified chamber. The breast normal cell line MCF-10A, provided by Professor Simran Tandon of Amity University, Noida, was maintained in DMEM/F12 (Invitrogen, USA) growth medium supplemented with 5% FBS, 1% antibiotic (Pen/strep) (Invitrogen, USA), and growth factors (20ng/mL EGF, 0.5mg/mL Hydrocortisone, 10g/mL Insulin, Sigma, USA). Cells were trypsinized (Trypsin, EDTA; Sigma, USA) every 4-7 days for experimental purposes and reseeded in fresh culture flasks [23].

### MTT assay

The efficacy of resveratrol was assessed on MCF-10A breast normal cell line, MCF-7, T-47D & MDA-MB-231 breast cancer cell lines by MTT assay (Sigma, USA) and stained with MTT dye (Sigma, USA). A 100mM working stock was prepared and treated with a different concentrations in µM. MTT assay was performed in a 96-well plate; cells were counted using a hemocytometer with an equal volume of trypan blue dye. 5,000 cells seeded in each well of 96 well plates and kept in an incubator overnight, next day treated with different concentrations (10-50µM) of resveratrol and incubated for 24 hours. then 20µL MTT dye (5mg/mL) was added to each well and incubated for 4 hours after that lysis solution was added, mixed well and incubated for 1 hours again finally read at 570nm wavelength by a microplate reader. From absorbance values calculated the percentage of viable cells [24].

### Consensus CpG rich promoter sequence retrieves

Consensus CpG rich promoter sequence retrieves promoter sequence of *BRCA1* and *BRCA2* was retrieved from the eukaryotic promoter database and a high CpG island was identified by EMBOCpG plot online tools, more than 40% bases were considered for promoter sequence retrieval for EMSA analysis. After submitting the promoter sequence to Tf sites scan online software (Table 1A), the consensus transcription factor binding region on these CpG islands was identified, and the high transcription factor binding site of this methyl-CpG sequence was used for EMSA analysis as forward and reverse probe for *BRCA1, BRCA2,* and *p16* genes [25].

**Table 1:** List ofprobe and primer sequence of BRCA1, BRCA2 & p16 gene used in EMSA for binding assay andChIP&MeIP assay for amplification of these genes and RT primer for the real time expression of MBD1, MBD2, MeCP2, BRCA1, BRCA2 & p16 gene

**Name**	**Sequences 5’-3’**
**(A)** **EMSA probe**	
BRCA1 (35bp) -229 to -194	Forward-5’-GCGTAGAGGCGAGAGGGCGGGCGCTTTACGGCGAA-3’ Reverse-5’-TTCGCCGTAAAGCGCCCGCCCTCTCGCCTCTACGC-3’
BRCA2 (37bp) -169 to -132	Forward-5’-TGCGTGTCGCGTCACGGCGTCACGTGGCCAGCGCGGG-3’ Reverse- 5’-CCCGCGCTGGCCACGTGACGCCGTGACGCGACACGCA-3’
P16 (38bp) -305 to -267	Forward-5’-CCCGAGCGGCCGGAACGAGGCGCGGAGCCGCGCTCCGG-3’ Reverse-5’-CCGGAGCGCGGCTCCGCGCCTCGTTCCGGCCGCTCGGG-3’
**(B) Un-methylated primers (ChIP assay)**	
BRCA1 (86bp)	Forward-5’ - TTG GTT TTT GTG GTA ATG GAA AAG TGT - 3’ Reverse-5’ - CAA AAA ATC TCA ACA AAC TCA CAC CA - 3’
BRCA2 (60bp)	Forward-5’-AGGGTGGTTTGGGATTTTTAAGG-3’ Reverse-5’-TCACACTTCTCCCAACAACAACC-3’
p16 (151bp)	Forward-5’- TTA TTA GAG GGT GGG GTG GAT TGT- 3’ Reverse-5’-CAA CCC CAA ACC ACA ACC ATA A - 3’
**(C) Methylated primers (MeIP assay)**	
BRCA1 (75bp)	Forward-5’ - TCG TGG TAA CGG AAA AGC GC – 3’ Reverse-5’ -AAATCTCAACGAACTCACGCCG-3’
BRCA2(62bp)	Forward-5’GACGGTTGGGATGTTTGATAAGG 3’ Reverse-5’-AATCTATCCCCTCACGCTTCTCC-3’
p16(150bp)	Forward-5’- TTA TTA GAG GGT GGG GCG GAT CGC -3’ Reverse-5’- GAC CCC GAA CCG CGA CCG TAA -3’
**(D) RT Primer**	
MBD1(107bp)	Forward 5’-CCTGGGTGCTGTGAGAACTGT-3’ Reverse 5’-TTGAAGGCAATTCTCTGTGCTC-3’
MBD2 (86bp)	Forward 5’-AGTGAAATCAGACCCACAACGAA-3’ Reverse 5’-CATCTGATGCACTAAGTCCTTGTAGC-3’
MeCP2(225bp)	Forward 5’- GCCTCTTTCCCTTCCAGTTTAT-3’ Reverse 5’-ACTGACTCGTGATGCCTTTG-3’
BRCA1(107bp)	Forward5’- ACAGCTGTGTGGTGCTTCTGTG-3’ Reverse 5’- CATTGTCCTCTGTCCAGGCATC-3’
BRCA2(350bp)	Forward 5’- CTTGCCCCTTTCGTCTATTTG-3’ Reverse 5’- TACGGCCCTGAAGTACAGTCTT-3’
P16(180bp)	Forward 5’- GCTGCCCAACGCACCGAATA-3’ Reverse 5’-ACCACCAGCGTGTCCAGGAA-3’
GAPDH(147bp)	Forward 5’-GACCACTTTGTCAAGCTCATTTC-3’ Reverse 5’-CTCTCTTCCTCTTGTGCTCTT-3’

### Electrophoresis mobility shift assay (EMSA)

Methylation of promoter sequence was done by using S-adenosine methionine and SSSI methylase (4U/µL) enzyme (BioLabs) using 10µM/µL DNA probe followed by 3’end Biotin labeling by 3’-end labeling kit (ThermoScientific). Binding studies of *MBD1, MBD2,* and *MeCP2* proteins on Methyl-CpG rich promoter region of *BRCA1, BRCA2* & *p16* genes in MCF-10A breast normal cells and MCF-7, T-47D, and MDA-MB-231 breast cancer cell lines was performed on 8% PAGE gel along with EBNA DNA and extract as a control. Samples were prepared according to the instructions given in Thermo scientific EMSA kit [26–28].

### ChIP assay

Chip assay was performed using the Abcam cross-linking chip assay (X-Chip) kit. MCF-10A, MCF-7, T47D & MDA-MB-231 cells cultured and after cross-linking proteins to DNA with formaldehyde, lysis and sonication were performed to shear DNA to an average fragment size of 200 - 1000 bp. After sonication, pelleted cell debris was removed, and the supernatant was used for further analysis. Primary antibodies were added to all samples except the control and rotate at 4°C for 1 hour. After blocking centrifuged the immuno-precipitated samples and were washed with wash buffer. Eluted DNA by adding elution buffer and DNA samples were used to perform PCR using *BRCA1* (86bp), *BRCA2* (60bp), and *p16* (151bp) genes forward and reverse primers (Table 1B) [29, 30].

### MeIP assay

This MeIP assay was performed by using methylation immuno-precipitation assay kit (Abcam) and following its protocols. Cell pellets were prepared by centrifugation, then sonicated, and the supernatant was collected. Immunoprecipitation was done using 5mC primary antibody following the kit’s protocol. Eluted DNA samples were used to perform PCR using *BRCA1* (75bp), *BRCA2* (62bp) and *p16* (150) forward and reverse primers (Table 1C) [31, 32].

### Real-time PCR

RNA isolation was done by the TriZol method and cDNA was prepared using cDNA synthesis kit (Thermofisher Scientific). Real-Time quantitative PCR of *MBD1, MBD2, MeCP2, BRCA1, BRCA2, p16* & *GAPDH* gene were performed using SYBR green master mix, RT primers (Table 1D), nuclease-free water, and 1µL of 100ng cDNA from MCF-10A breast normal and MCF-7, T-47D, MDA-MB-231 breast cancer cell line untreated and treated with different concentrations (10-50μM,) of resveratrol. CT values were generated of each gene and analyzed using the relative quantification method and fold change was shown in the bar graph [33].

### Western blotting

MCF-10A, MCF-7, T-47D & MDA-MB-231 cell lines were cultured and an appropriate number of cells seeded in a 6-well plate for 80-90% confluence in 24 hours. Different concentrations of resveratrol (10-50μM,) were treated to the cells over 24 hours and protein was extracted by using RIPA buffer containing protease inhibitors and quantified. SDS- polyacrylamide gel electrophoresis was done to separate proteins according to molecular size and their electrophoretic mobility using tris-glycine running buffer. 10% of SDS page gel was prepared according to the molecular size of protein and run on tris-glycine buffer containing SDS and transferred in PVDF membrane by western blotting using tris-glycine and 10-20% methanol, protein-specific primary antibody (Abcam;-*MBD1* (ab2846), *MBD2* (ab38646), *MeCP2* (ab2828), *BRCA1* (ab191042), *BRCA2* (ab123491), *p16* (ab81278) & Sigma *β-Actin* (A1978) ) were used to bind protein band and HRP conjugated secondary antibody was used to detect the band after adding the luminal and exposed to X-ray film. We have used small cut piece of x-ray film to exposed PVDF membrane in the dark room to detect bands and all processed images included in the results and original unprocessed separately in Supplementary file [34].

### Clonogenic assay

MCF-10A, MCF-7 & MDA-MB-231 cell lines were cultured, trypsinized and single cells were made by pipetting the medium. Cells were counted and 1000 cells were seeded into 6-well plates. Incubated cells for a few hours in a CO_2_ incubator at 37°C and allow to adhere on the plate/dish. Cells were treated with different concentrations (5μM, 10μM, 15μM, 20μM, 25μM, 30μM) of resveratrol. Incubated the cells for 1-3 weeks until cells in control plates have formed colonies that are of a substantially good size 50 cells per colony are the minimum for scoring. Removed the media and fixed the cells with 2-3mL of fixation solution (4:1 Methanol/Acetic acid) and kept at room temperature for 10min. Stained the cells with 0.5% crystal violet staining solution for 2 hours at room temperature and immersed the dishes/plates in tap water to rinse off crystal violet. Air-dried the dishes/plates on a table cloth at room temperature and images were captured. Counted number of colonies with Image-J software. Calculate plating efficiency (PE) and surviving fraction (SF). PE = No. of colonies formed/ No. of cells seeded x 100% SF = No. of colonies formed after treatment/ No. of cells seeded x PE [35].

### Migration assay

MCF-10A, MCF-7 & MDA-MB-231 cell lines were cultured and an appropriate number of cells seeded in 6-well plates for 100% confluence within 24 hours. In a sterile environment typically a biosafety hood, used a 200μl pipette tip to press firmly against the top of the tissue culture plate and swiftly make a vertical wound down through the cell monolayer. Carefully aspirated the media and cell debris from plates. Slowly added enough culture media against the wall of the well to cover the bottom of the well to avoid detaching additional cells. Wound closure was monitored and photographs were captured. At various time intervals, such as every 6 hours, examined it under an inverted microscope for wound closure. Wound closure duration varies depending on the cell type, therefore Image J software was used to evaluate the findings of snapshot photographs, quantified the distance of wound closure with a scale bar, and depicted wound closure over time with a line plot or bar graph [36].

### Sphere formation assay

Ultra-low adherent plates were used for the sphere formation experiment. Cell lines were grown in media containing 10% FBS and 1% antibiotics at 37^0^C and 5% CO_2_ in an incubator and counted using a hemocytometer. 2000 cells were seeded in each well of 96 well plates with 200μl serum-free medium and a different dose of resveratrol to each well of the ultralow adherent plate (BD Bioscience) and incubated in an incubator at 37^0^C and 5% CO_2_. The next day, photographs of the spheres were taken, and the size of the spheres was measured on the first and fifth day from the day of cell seeding, and a bar graph was created [37–39].

### Apoptosis assay (Acridine Orange/ EtBr method)

MCF-10A, MCF-7, T-47D & MDA-MB-231 cell lines were cultured and cells counted using hemocytometer. 5 X 10^3^ cells were seeded in a 96-well plate and incubated for 24 hours so that the cells get to adhere to the surface of the wells. Also, MCF-7, T-47D & MDA-MB231 cell line’s spheres were generated in an ultralow adherent plate using serum-free media. Different concentrations (10μM, 20μM, 30μM, 40μM, 50μM,) of resveratrol containing media was added to the cells and incubated at 37^0^C with 5% CO_2_ for 24 hours. Then media was removed and cells were fixed using 4% PFA for 30 minutes and washed with 1x PBS followed by staining with 1mg/mL AO/EtBr (Sigma, USA) staining dye for 10 minutes and again washed with 1x PBS. An inverted fluorescent microscope with a 488nm wavelength filter was used to record pictures of cells monolayer and spheres [40].

### Statistical analysis

All data were statistically analyzed by Prism5 software. Values in text and figures are presented as mean ± SEM unless otherwise stated. Statistical significance was determined by unpaired t test or one-way ANOVA. The *P* values <0.05 were considered significant.

## Results

### Cell culture and MTT assay

**Fig. 1:**
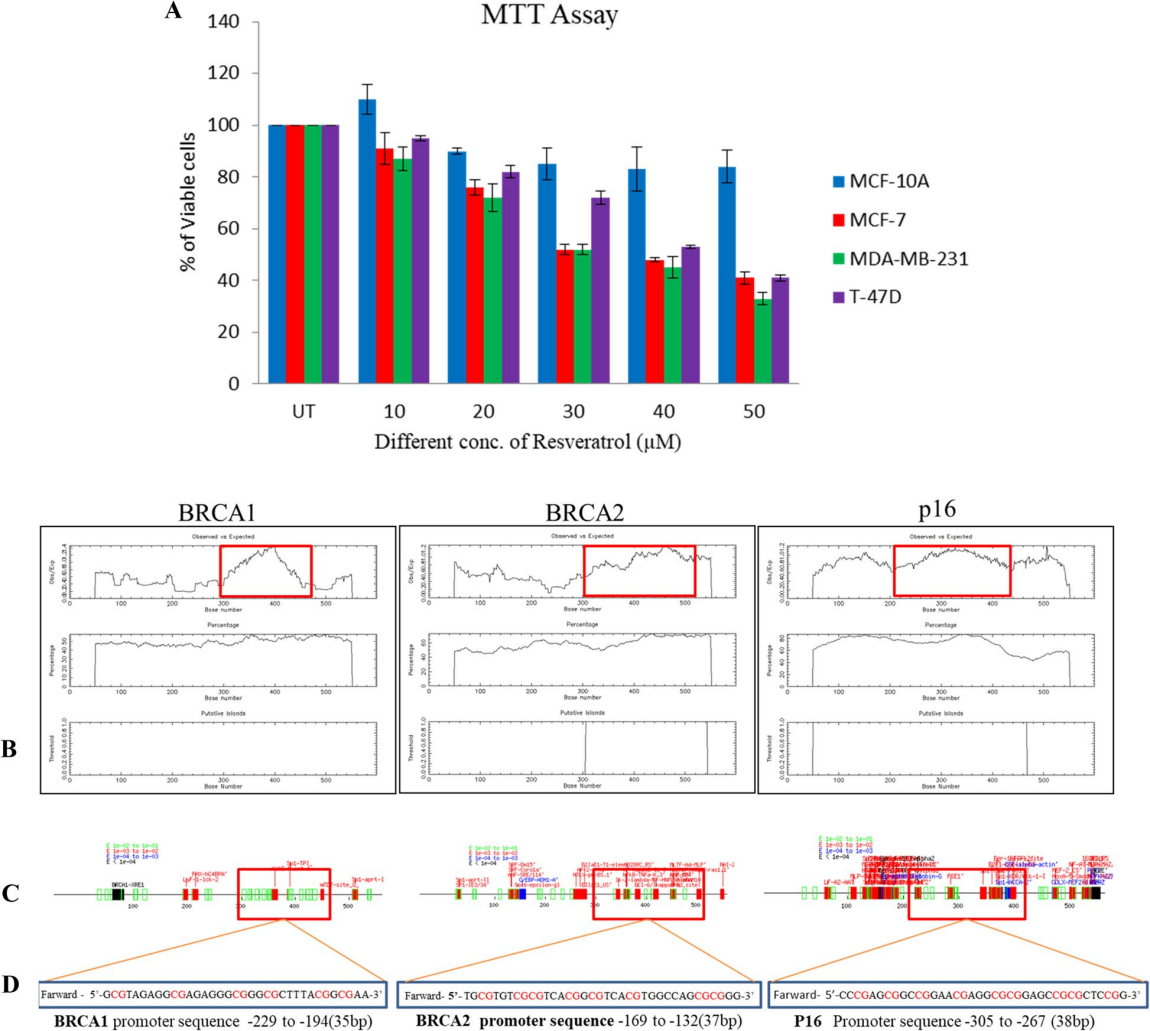
**A** MTT assay of Resveratrol was done on MCF-10A, MCF-7 , MDA-MB-231 & T47D Breast normal and cancer cells. % of viable cells Vs. Resveratrol concentration calculated and plotted as histogram. **B-D** High CpG rich and transcription factor binding site Promoter sequences of *BRCA1, BRCA2 & p16* gene was identify by Insilico using CpG plot and Tfscan online database tool and used for EMSA analysis as biotin labeled probe for protein binding

Cell lines, including MCF-10A, MCF-7, T-47D, and MDA-MB-231 were cultured and maintained in DMEM-F12 and DMEM media with FBS and antibiotics at 37^0^C with 5% CO2. MTT assay was performed in these breast normal and cancer cell lines using Resveratrol. After seeding 10,000 cells in each well of 96 well plates and incubated overnight, cells were treated with different concentrations (10-50μM) of Resveratrol. MTT dye (5mg/mL) was added to each well and incubated for 4 hours followed by lysis solution was added and incubated for another 1 hour before being read at a wavelength of 570nm. From the absorbance, calculated the percentage of viable cells in these cell lines and found that 30μM concentration of Resveratrol gives 50% viable cells. MCF-10A cell line showed no effect of resveratrol up to 50μM concentration (Fig. 1A).

### Consensus CpG rich promoter sequence retrieved by database by Insilco methods

The promoter sequences of *BRCA1, BRCA2* and *p16* were retrieved from the eukaryotic promoter database. A high CpG island was identified in these promoter sequences using the EMBOCpG plot. Bases with more than 40% of CpG sequences were considered for CpG island promoter sequence for EMSA analysis. The Tf sites scan tool was used to identify the high transcription factor binding site of this methyl-CpG promoter sequence. A 35-38 base sequence from high transcription factor binding site of this methyl-CpG sequence taken for EMSA analysis as forward and reverse probe for *BRCA1, BRCA2* & *p16* genes (Fig. 1B-D).

### MBD proteins bind to the promoter sequence of the BRCA1 gene in breast cancer cells

**Fig. 2:**
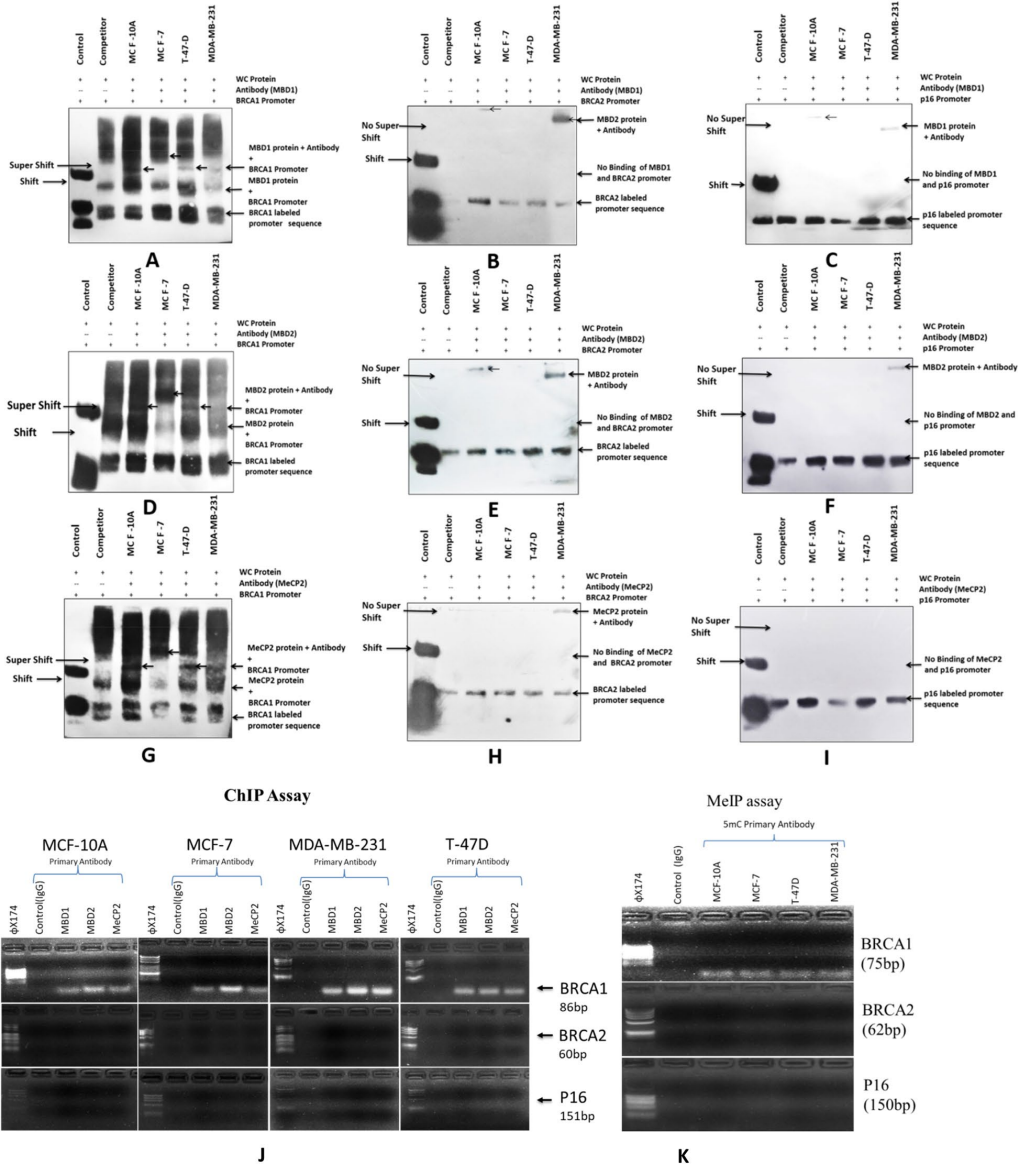
**A-I** Promoter binding of *MBD1, MBD2 & MeCP2* proteins on *BRCA1, BRCA2 & p16* genes were analyzed by EMSA assay on 8% PAGE gel and transferred in nylon membrane then exposed in X-ray film. Shifting of bands was observed by using protein specific primary antibody arrow showing in between image indicate the bands. **J** Chromosome immune precipitation of *BRCA1, BRCA2 & p16* gene was done by ChIP assay using *MBD1, MBD2 & MeCP2* primary antibody and amplification of these genes were done by PCR and run on agarose gel and bands was observed. **K** Methylation immune precipitation of *BRCA1, BRCA2 & p16* genes were done by MeIP assay using 5mC methylation specific primary antibody and amplification of these genes were done by PCR and run on 10% agarose gel to observed bands

EMSA was performed to check the DNA binding activity of MBD proteins on *BRCA1, BRCA2*, and *p16* gene promoter in breast cancer. We performed EMSA analysis using biotin labeled promoter sequence of *BRCA1, BRCA2*, and *p16* gene retrieved from EMBOCpG plot for *MBD1, MBD2,* and *MeCP2* proteins binding isolated from MCF-10A, MCF-7, T-47D & MDA-MB-231 human breasts normal and cancer cell line. EMSA results revealed the presence of active MBD proteins binding on *BRCA1* promoter sequence in all cell lines using a specific primary antibody. Shift of bands, as well as a supershift of *MBD1, MBD2* & *MeCP2*, proteins and *BRCA1* promoter along with protein specific primary Antibody complexes in an PAGE gel, confirmed the MBD proteins binding on *BRCA1* promoter sequence in MCF-10A, MCF-7, T-47D & MDA-MB-231 breasts normal and cancer cell line but no binding was observed on *BRCA2* and *p16* promoter sequences as there was no bands observed on gel in the cell lines mentioned above (Fig. 2A-I).

### BRCA1 gene was identified by ChIP assay in immuno-precipitation complex in breast cancer cells

ChIP assay was performed to confirm MBD proteins binding on *BRCA1, BRCA2*, and p16 gene promoters in MCF-10A, MCF-7, T-47D & MDA-MB-231 cell lines using ChIP assay kit. First cross-linked the proteins with DNA using formaldehyde and sonicated to breakdown the DNA into the fragments. Precipitated the bound DNA using *MBD1, MBD2* & *MeCP2* proteins specific primary antibody which was amplified by PCR using forward and reverse primers and found that only *BRCA1* was amplified by PCR and bands were observed in the agarose gel but no amplification was observed for *BRCA2* and *p16* genes which confirm MBD proteins binding on *BRCA1* gene promoter sequence (Fig. 2J).

### MeIP assay identified BRCA1 gene presence in the methylated immune precipitation complex in breast cancer cells

The Abcam MeIP test kit was used to perform the methylation immune precipitation experiment using a 5mC primary antibody. DNA isolated from MCF-10A, MCF-7, T-47D & MDA-MB-231 cell lines and precipitated by 5mC primary antibody. Precipitated DNA was used to amplification of *BRCA1*, *BRCA2*, and *p16* genes using forward and reverse primers and after running PCR product in agarose gel we found that only the *BRCA1* gene was amplified by PCR but no amplification was observed for *BRCA2* and *p16* genes. This demonstrated that the *BRCA1* promoter sequence was substantially methylated, as shown by the presence of a 5mC primary antibody (Fig. 2K).

### MBD proteins regulate the BRCA1 gene expression in resveratrol treated brest cancer cells

**Fig. 3:**
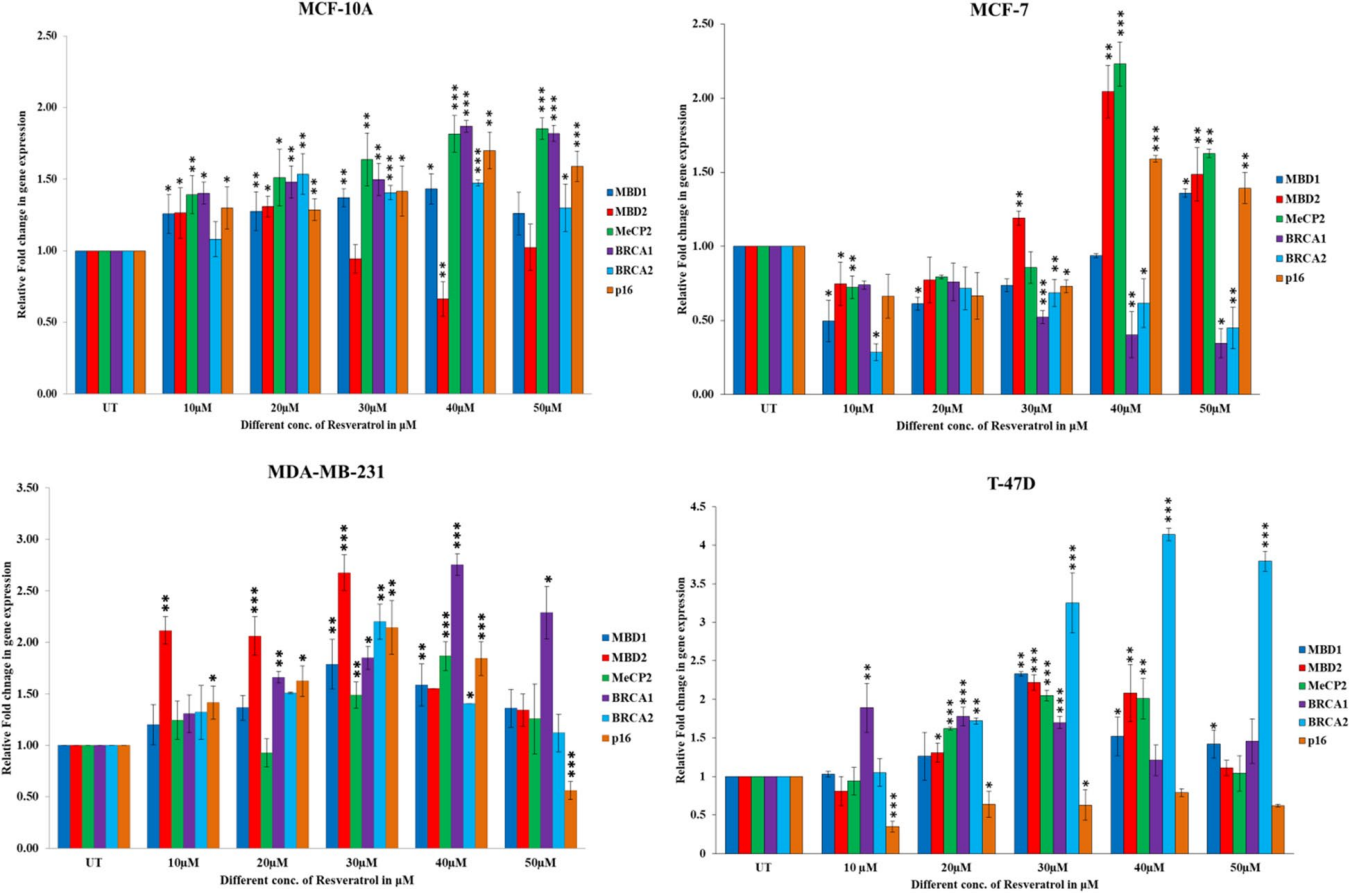
Real time gene expression analysis of *MBD1, MBD2, MeCP2, BRCA1, BRCA2 & p16* gene were done in MCF-10A, MCF-7, MDA-MB-231 & T-47D breast normal and cancer cell lines treated with different concentration of resveratrol. CT value of each gene was calculated by using relative quantitative methods and normalized with housekeeping *GAPDH* gene and plotted as bar graph

Real-time gene expression analysis was done to check gene expression in correlation with resveratrol treatment. We have performed Real-time PCR of *MBD1, MBD2, MeCP2, BRCA1, BRCA2, p16*, and *GAPDH* genes, in MCF-7, MDA-MB-231 & T-47D breast cancer, and MCF10A breast normal cell lines treated with different concentrations of resveratrol. *MBD1, MeCP2, BRCA1, BRCA2*, and *p16* genes expression were up-regulated in MCF-10A cells with increasing concentrations of resveratrol, with showing extremely significant results at 40µM (*BRCA1* t-test *P*<0.0007, One way ANOVA *P*<0.00018; *p16* t-test *P*<0.0045, One way ANOVA *P*<0.0006). *MBD2* gene expression was down regulated along with increasing concentrations of resveratrol in the MCF-10A breast epithelial cell line. *MBD2, MeCP2*, and *p16* genes expression were up-regulated with increasing resveratrol concentrations in the MCF-7 cell line, with the highest levels at 40µM (*MBD2* t-test *P*<0.0124, One-way ANOVA= *P*<0.0001, *MeCP2* t-test *P*<0.0007, One-way ANOVA= *P*<0.0001 and *p16* t-test *P*<0.0001, One-way ANOVA= *P*<0.0001). However, at 50µM, the expression of *BRCA1* (t-test *P*<0.0133, One-way ANOVA=*P*<0.0227) & *BRCA2* (t-test *P*<0.0025, One-way ANOVA=*P*<0.0043) genes were down-regulated and significant. In the MDA-MB-231 cell line, *BRCA1* gene expression was up regulated along with the increasing concentrations of resveratrol and has a significant high at 40µM (t-test *P*<0.0001, One-way ANOVA=*P*<0.0371) concentration. *MBD1* (*P*<0.0047) *MBD2* (*P*<0.0001), *BRCA2* (*P*<0.0044) genes were up-regulated up to 30µM and *MeCP2* (*P*<0.0004), & *p16* (*P*<0.0008) up to 40µM then down-regulated. However in T-47D cell line *MBD1* (*P*<0.0023), *MBD2* (*P*<0.0001), *MeCP2* (*P*<0.0001) genes expression up-regulated up to 30µM and *BRCA2* (*P*<0.0001) gene up to 50µM concentration and has significant at this concentration, whereas *BRCA1* & *p16* genes expression is down-regulated along with the increasing concentrations of resveratrol (Fig. 3).

Correlation analysis also revealed that in MCF-7 *BRCA1* & *BRCA2* negatively correlated with resveratrol treatment and *BRCA1* has significant (Pearson r = -0.9581, *p*<0.0026). In MDA-MB-231 all genes are positively correlated except *p16*, whereas *BRCA1* significantly positively correlated (Pearson r = 0.9257, *p*<0.0081) to resveratrol. *BRCA2* significantly positively correlated in T-47D (Pearson r = 0.9366, *p*<0.0059) breast cancer cells and *BRCA1* & *p16* negatively correlated but not significant. However in MCF-10A breast normal cell line all gene except *MBD2* has positively correlation, out which *MeCP2* (Pearson r = 0.9380, *p*<0.0056), *BRCA1* (Pearson r = 0.9338, *p*<0.0064) and *p16* (Pearson r = 0.9225, *p*<0.0088) have significantly correlated to resveratrol treatment.

### MBD proteins expression and their regulation on the BRCA1 protein expression in resveratrol treated breast cancer cells

**Fig. 4:**
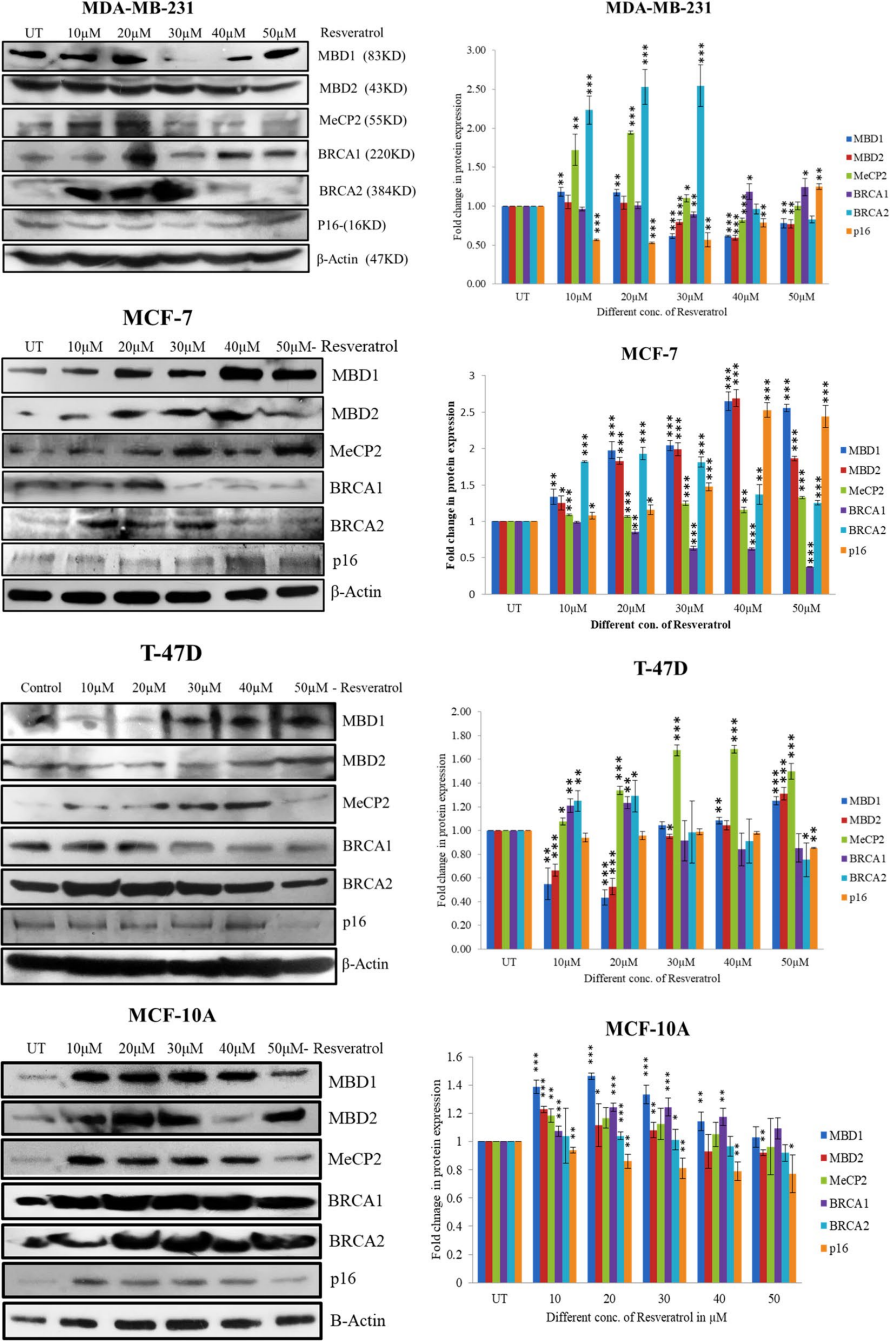
Protein expression of *MBD1, MBD2, MeCP2, BRCA1, BRCA2 & p16* genes were done by western blotting in resveratrol treated MDA-MB-231, MCF-7, T-47D & MCF-10A breast normal and cancer cell lines. Bands were transferred to PVDF membrane and exposed to X-ray film. Densitometry analysis was done by MyImage analysis software (thermo Scientific) to quantify the bands intensity and normalized with housekeeping β-actin gene protein and bar graph plotted as fold change in protein expression vs. concentration of resveratrol

Western blotting analysis was performed In MCF-7, MDA-MB-231, T-47D breast cancer, and MCF10A breast normal cell lines, to check protein expression of *MBD1, MBD2, MeCP2, BRCA1, BRCA2, p16*, and *β-actin* genes in relation to resveratrol treatment. We discovered that in MCF-7 cells *MBD1, MBD2, MeCP2* & *p16* protein expression were up-regulated and significant at 40µM (*P*<0.0001) and *BRCA1* protein expression down regulated with increasing concentrations of resveratrol and significant at 50µM (*P*<0.0001) concentration. Also *BRCA2* protein expression down regulated up to 50µM concentration but not significant. ANOVA analysis also showed that change in protein expression was significant *P*<0.0001 concerning resveratrol treatment in all genes. In MDA-MB-231 breast cancer cells *MBD1* (*P*<0.0001), *MBD2* (*P*<0.0003), *MeCP2* (*P*<0.0003), protein expression were down-regulated and significant at 40µM and *BRCA2* at 50µM (*P*<0.0033) concentration of resveratrol. Whereas *BRCA1* (*P*<0.0172) and *p16* (*P*<0.0003) protein expression were up-regulated and significant at 50µM concentration of resveratrol. Also ANOVA analysis revealed that all gene showed significant* P*<0.0001 change in protein expression. In T-47D cell line *MBD1, MBD2, MeCP2* (40µM* P*<0.0001) protein expression up-regulated and significant at 50µM for *MBD1* (*P*<0.0002) & *MBD2* (*P*<0.0005), whereas *MeCP2* at 40µM (*P*<0.0001). *BRCA1, BRCA2* and *p16* protein expression down-regulated along with increasing concentrations of resveratrol and significant at 50µM concentration. ANOVA analysis in T-47D cells though showed significant but only *MBD1* & *MBD2* genes have highly significant *P*<0.0001 change in protein expression concerning resveratrol. In the MCF-10A protein expression is upregulated up to 30µM of resveratrol treatment, however, there are no significant effects of resveratrol on protein expression of *MBD1, MBD2, MeCP2, BRCA1, BRCA2, p16* genes observed in MCF-10A cell breast normal cell line (Fig. 4).

The association between protein expression and resveratrol levels was also investigated using correlation analysis. *MBD1* (Pearson r 0.9626, *P*<0.0021), *MeCP2* (Pearson r 0.8903, *P*<0.0174), and *p16* (Pearson r 0.9131, *P*<0.0110) were substantially favorably connected with resveratrol therapy in MCF-7 cells, whereas *BRCA1* (Pearson r -0.9683, *P*<0.0015) was adversely correlated. We have also found a negative correlation between MBD proteins and *BRCA1* protein expression in MCF-7 cells where *MBD1* & *MeCP2* has significant negative correlation with *BRCA1* but *MBD2* not significant negative correlation. In MDA-MB-231 cells *MBD1, MBD2, MeCP2* & *BRCA2*, negative correlated to resveratrol and *BRCA1* & *p16* positively correlated with resveratrol treatment. We have also checked the correlation between MBD proteins and *BRCA1* protein expression and found that it is negatively correlated but not significant. In the T-47D cell line only *MeCP2* (Pearson r 0.8449, *P*<0.0342) positively correlated to resveratrol treatment and there is a negative correlation between MBD proteins and *BRCA1* protein expression and *MBD1* (Pearson r -0.9688,* P*<0.0014) & *MBD2* (Pearson r -0.9153, *P*<0.0105) has significant correlation but *MeCP2* not significantly correlated.

### Resveratrol inhibited proliferation and colony formation in breast cancer cells

**Fig. 5:**
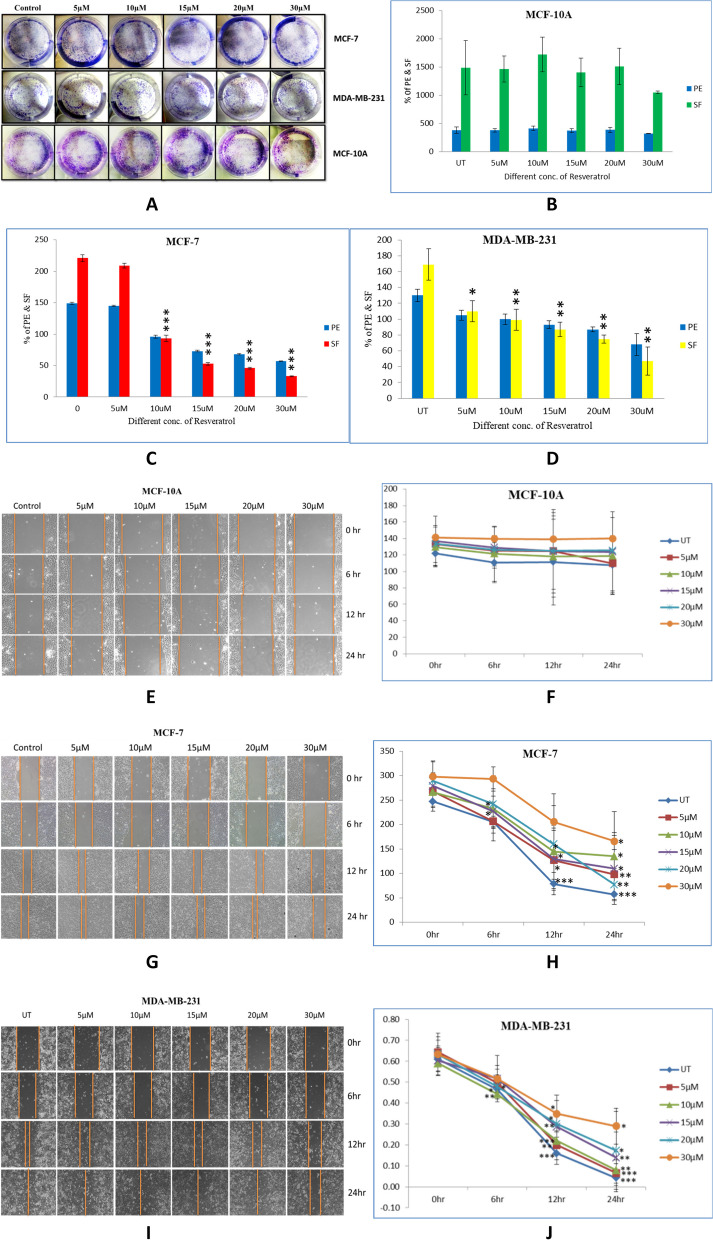
**A-D** Clonogenic assay of MCF-10A, MCF-7 & MDA-MB-231 cells treated with different concentration of resveratrol was done on 6 well plates and stained with crystal violet after fixing the cells and images were captured using scale bar (10X resolution, 2.3mm) in fluorescent inverted microscope. Colonies were counted by Image J software and their plating efficiency and survival frequency was calculated using formula and plotted as bar graph. **E-J** Migration assay was done by wound healing assay on MCF-10A, MCF-7 & MDA-MB-231 cells treated with different concentration of resveratrol and images were captured using scale bar (10X resolution, 2.3mm) in fluorescent inverted microscope and gap of wound closure was calculated by Image J software and plotted as bar graph

Clonogenic assay was performed to elucidate colony formation capacity of MCF-10A, MCF-7 & MDA-MB-2321 cells after resveratrol treatment. Cells were counted and 1000 cells were seeded in each well of 6 well plates and allowed cells to adhere on the surface. Cells were treated with different concentrations of resveratrol (5µM, 10µM, 15µM, 20µM, 30µM) and allowed cells to grow until two colonies keep minimum distance in control sample. Media was removed and cells were fixed with fixation solution (Methanol & Glacial Acetic acid 7:1) for 30 minutes and stained with crystal violet for 30 minutes again and washed with tap water and let it dry (Fig. 5A). Images were captured and colonies were counted using Image-J software and survivals frequency was calculated using the formula. From the above results we discovered that survival frequency decreased as resveratrol concentration increasing in MCF-7 (One-way ANOVA *P*<0.0001) and MDA-MB-231 (One-way ANOVA *P*<0.0001) breast cancer cell lines, with 50% survival at 20µM (t-Test *P*<0.0001) and 30µM (t-Test *P*<0.0015) respectively, but no effect observed on MCF-10A breast normal cell line (Fig. 5B-D).

### Resveratrol inhibited the migration of breast cancer cells at a high concentration

To confirm the anti-metastatic property of resveratrol in breast cancer cells. We have performed migration assay also called wound healing assay in MCF-10A, MCF-7 & MDA-MB-231 Cell lines. Cells were cultured and enough cells were seeded in each well of 6 well plates. After 100% confluence, media was removed and washed with 1x PBS, the scratches were done using pipette tips and washed out. Different concentrations (5µM, 10µM, 15µM, 20µM, 30µM) of resveratrol were treated to each well, and images of the scratches were taken every 6 hours from 0-24 hours time duration and migration distances were measured and calculated. We observed that after 24 hours treatment, migration of MCF-7 (*P*<0.0271) and MDA-MB-231(*P*<0.0111) cell lines were reduced with the increasing concentration of resveratrol and significant at 30µM concentration, and no migration was observed on MCF10A breast normal cell line (Fig. 5E-J).

### Resveratrol inhibited the sphere formation of breast cancer cells

**Fig. 6:**
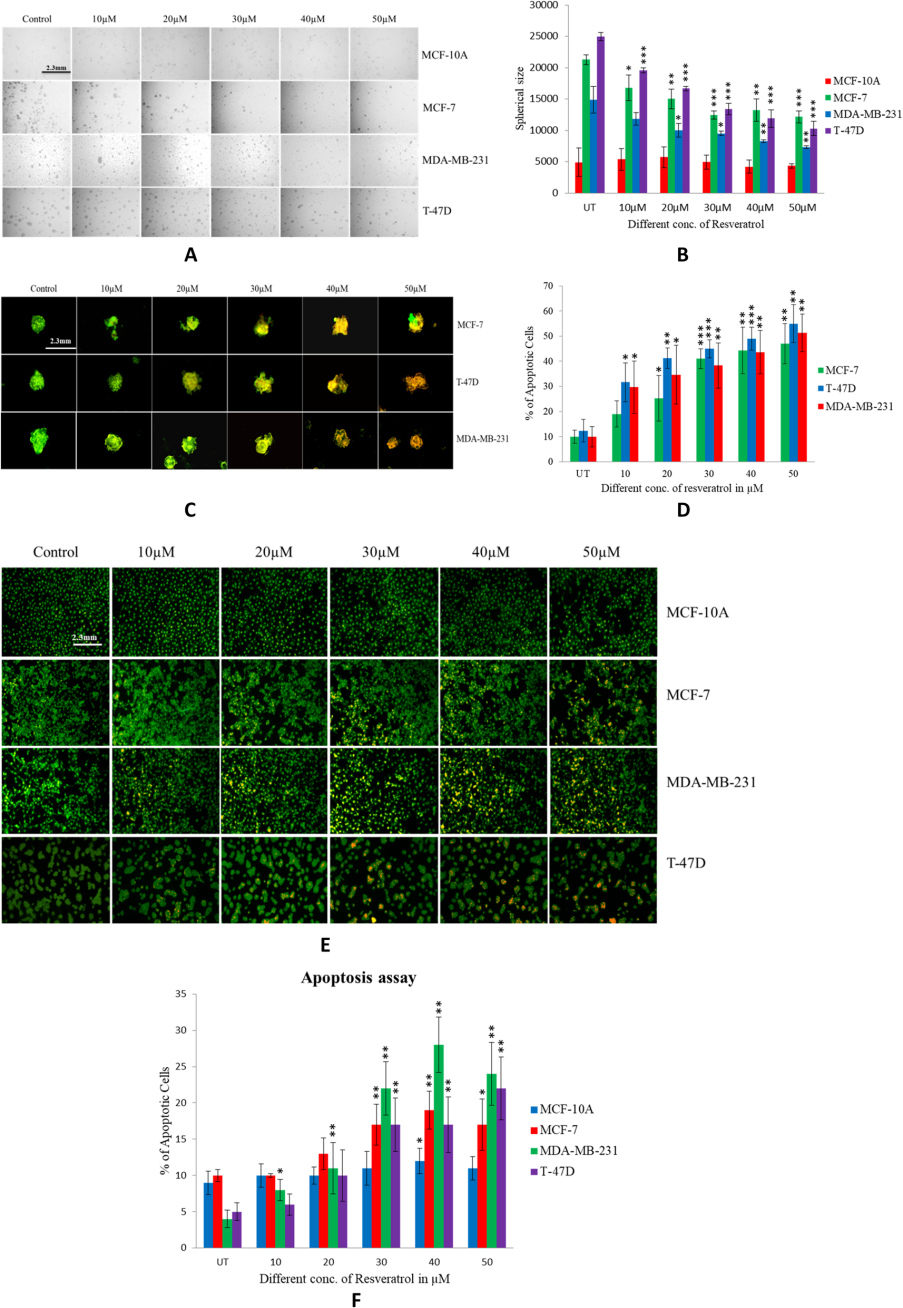
**A-B** Sphere formation assay of MCF-10A, MCF-7, MDA-MB-231& T-47D breast normal and cancer cell line were done on 96 well plates treated with different concentration of resveratrol and Images were taken at scale bar (10X resolution, 2.3mm) by inverted fluorescence microscope and spheres size were calculated by imaging software (Rad scientific) and presented graphically as bar graph as sphere size vs. resveratrol concentration. **C-D** Sphere of MCF-7, MDA-MB-231 & T-47D cells were treated with different concentration of resveratrol and apoptotic cells were stained with acridine orange/Etbr staining dye. Images were taken at scale bar (10X resolution, 2.3mm) by inverted fluorescence microscope and % of apoptotic cells were counted using Image-J software and graphically presented by bar graph. **E-F** Apoptosis assay was performed onMCF-10A, MCF-7, and MDA-MB-231 & T-47D breast normal and cancer cells treated with different concentration of resveratrol. Images were taken at scale bar (10X resolution, 2.3mm) by inverted fluorescence microscope and apoptotic cells were counted using Image-J software and graphically presented by bar graph as % of apoptotic cells vs. resveratrol concentration

Sphere formation assay was performed to demonstrate that the size of spheres in MCF-10A, MCF-7, and MDA-MB-231 & T-47 D Cell lines treated with increasing concentrations of resveratrol. We have performed sphere formation assay in an ultralow adherent plate with serum-free media containing antibiotics along with the different concentrations of resveratrol and we found that at 5^th^-day sphere size of MCF-7, MDA-MB-231 & T-47D (One-way ANOVA:* P*<0.0001) cells were reduced along with the increasing concentrations of resveratrol and have significant at 50µM concentration (t-test; MCF-7 *P*<0.0002, MDA-MB-231* P*<0.0036, T-47D* P*<0.0001). No sphere formation was observed in MCF-10A breast epithelial cells (Fig. 6A-B).

### Resveratrol increased apoptotic cells no. in the sphere at high concentration in breast cancer cells

To perform apoptosis in sphere model, we have treated spheres of breast cancer cells with different concentrations of resveratrol and stained them with acridine orange/ EtBr staining dye after 24 hours. We observed apoptotic cells in sphere by fluorescence microscope and found that higher concentrations of resveratrol increasing the apoptotic cells in the spheres of breast cancer cells (One-way ANOVA: MCF-7 *P*<0.0001, T-47D *P*<0.0001; MDA-MB-231 *P*<0.0019) and significant at 30µM (*P*<0.0004) in MCF-7. In T-47D and MDA-MB231 30µM to 40 µM (*P*<0.0006), and 30µM (*P*<0.0076) to 50 µM (*P*<0.0011) significant respectively (Fig. 6C-D).

### Resveratrol increases apoptosis at high concentration in cells monolayer of breast cancer cells

The cytotoxicity of resveratrol was assessed by apoptosis analysis on MCF-10A, MCF-7, MDA-MB-231, and T-47D cells monolayers of breast normal and cancer cell line. We observed that after 24hr resveratrol treatment apoptotic cells number were increasing along with the increasing concentrations of resveratrol in MCF-7, MDA-MB-231 & T-47 D (One-way ANOVA: *P*<0.0010;* P*<0.0001; *P*<0.0002 respectively) breast cancer cell line and has significant at 40µM in MCF-7 (*P*<0.0021), and MDA-MB-231(*P*<0.0070), 50µM in T-47D (*P*<0.0032) breast cancer cell line, however, no effects was observed on MCF10A breast normal cells (Fig. 6E-F).

## Discussion

Today, epigenetics and its relevance in cancer research is a broad area of study [41]. DNA methylation and its many functions inside eukaryotic cells have been revealed [42]. In conjunction with histone modifications and other chromatin-associated proteins, it offers a stable gene silencing mechanism that regulates gene expression and chromatin remodeling [43]. *MBD1, MBD2,* and *MeCP2,* are directly involved in the transcriptional repression of methylated templates in vertebrates [22]. Resveratrol, a phytochemical present in grapes, berries, and peanuts, has been discovered to regulate *BRCA1* and *BRCA2* genes in breast cancer cells and has inhibitory effect on cellular events associated with tumor initiation, promotion and progression, according to previous research [17]. It has been shown that it exhibit anti-oxidative and anti-inflammatory and suppresses proliferation of several types of cancer such as colon, breast, pancreas, prostate, ovarian and endometrial cancer. Resveratrol binds to and activate the oestrogen receptors and aromatic hydrocarbon receptor that regulate the transcription of *BRCA1* genes [17, 44]. Resveratrol binds to the amino acid back bone of methyl binding domain and TRD domain of MBD proteins has been confirmed by docking and MD simulation method and reported in my previous study. Binding of resveratrol to methyl binding domain and TRD domain of MBD proteins will interfere with functioning of DNA binding and gene transcription [45]. This study begins by emphasizing the significance of epigenetics in cancer research, particularly focusing on DNA methylation and its role in stable gene silencing. *MBD1, MBD2*, and *MeCP2*, known players in transcriptional repression of methylated templates, are explored in the context of breast cancer. This study demonstrates the function of MBD proteins, which binds to and regulate the methyl promoter of *BRCA1, BRCA2*, and *p16* genes. This regulatory role of MBD proteins can be modulated by resveratrol. We began our research by calculating the IC50 of resveratrol on breast cancer cells and found that 30µM concentration of resveratrol inhibit 50% of cell viability of breast cancer cells. We demonstrated *MBD1, MBD2* & *MeCP2* proteins binding to the promoter region of the *BRCA1, BRCA2* and *p16* genes by EMSA assay using methylated promoter sequences and observed that these MBD proteins, specifically *MBD1, MBD2*, and *MeCP2*, bind to the promoter region of the *BRCA1* gene. Binding of *MBD2* is also reported to the methylated region of the gene promoter by Gunther et al., 2013 in their study which confirmed our findings [46]. However, MBDs protein binding with *BRCA2* and *p16* gene promoter was not observed in our study. Whereas Magdinier et al. confirmed *MBD2* binding in the *p16* gene promoter in colon cancer, which was revert by using 5aza-dc that clear its binding on methylation pattern, not on sequence specific [47]. Whereas Auriol et al. reported *MBD2* binding was gene specific in their experiments after hypo-methylation, so still it is not clear whether it is methylated specific or gene specific [22]. However we used methylated sequence in EMSA assay and observed that it’s binding on *BRCA1* gene promoter. We also used chromosome immune precipitation (ChIP) and methylation immune precipitation (MeIP) assays to confirm the binding of these MBDs proteins to the promoter regions of *BRCA1, BRCA2*, and *p16* genes, and discovered that only the *BRCA1* gene was amplified in precipitation, but no amplification was observed of *BRCA2* or *p16* gene, this results also supported by previous study [22]. Though previously it was reported that *MBD2* bind to the methylated promoter of the gene, whereas we have compared *MBD1, MBD2* and *MeCP2* binding on *BRCA1, BRCA2* and *p16* genes in this study by EMSA as well as ChIP assay. Based on above it is implying that *MBD1, MBD2*, and *MeCP2* bind only to the *BRCA1* gene promoter in ER+, PR+ & triple-negative breast cancer and control cell lines.

We have investigated real-time gene expression of *MBD1, MBD2* & *MeCP2* genes as well as *BRCA1, BRCA2* & *p16* genes in breast cancer cells treated with different concentrations of resveratrol and discovered that *MBD1, MBD2, MeCP2* and *p16* gene expression was up-regulated in MCF-7 cells and *BRCA1*(Pearson r = -0.9581, *P*<0.0026), *BRCA2* gene expression was down-regulated, however in MDA-MB-231 cell line except for *MBD2* all genes expression up-regulated in response to increasing concentrations of resveratrol, and *BRCA1* (Pearson r = 0.9257, *P*<0.0081) exhibits a strong positive correlation. In T-47D cell line *MBD1, MBD2*, *MeCP2* and *BRCA2* genes expression up-regulated and *BRCA1* & *p16* genes down-regulated. This indicates that the *MBD1* and *MeCP2* gene negatively regulates the *BRCA1* gene expression in ER+ and PR+ breast cancer cells and *MBD2* negatively regulates the *BRCA1* gene expression in ER+ & PR+ and Triple negative breast cancer cells after resveratrol treatment, however, there is no effects observed in genes expression in MCF10A breast normal cell line. Our gene expression study is also supported by earlier studies [17, 44]. However, *BRCA1* gene expression in MCF-7 cells showed down-regulation due to unknown region. Muller et al., 2003 reported the decreased expression of *BRCA1* in sporadic breast cancer [48]. Also dense methylation of CpG islands leads to down-regulation of *BRCA1* gene, probably mediated by methyl-CpG binding proteins reported by Magdinier et al., 2000 in their study [47]. However role of NuRD complex in the regulation of gene expression also reported by Wood et al. 2016, where it binds to *MBD2* and methylated regions and repress the gene expression. NuRD can interact to *MBD2b* and binds to the un-methylated region and regulate gene expression, these findings support the view that *MBD2*, through its interactions with NuRD, may be involved in transcriptional activation as well as repression [8]. Fustier et al. reported resveratrol causes up regulation of *BRCA1* gene expression but no change observed in protein expression, we have compared our results with this earlier reported study and found the difference could be due to long time exposure of resveratrol to MCF-7 cells for 48hr and also in their experiment author has used multiplex PCR with high annealing temperature 95°C and primer concentration 200nm, it could affect the expression result [17]. Whereas we have treated resveratrol for 24hr for 30µM and also we have studied the resveratrol binding on MBD proteins and it’s binding on *BRCA1* promoter. We observed that our protein expression results same as our gene expression, whereas Fustier et al., 2003 didn’t get protein expression results on their study [17].

Our western blotting results also showed that in MCF-7 & T-47D breast cancer cell *MBD1, MBD2* & *MeCP2* protein expression up-regulated and BRCA1, BRCA2 & p16 protein expression down-regulated significantly along with the increasing concentrations of resveratrol. In MDA-MB-231 cells *BRCA1* & *p16* protein expression up-regulated and *MBD1, MBD2, MeCP2* & *BRCA2* protein expression down-regulated. There is a negative correlation between MBD proteins & *BRCA1* protein expression in MCF-7 & T-47D cells which indicate that MBD proteins negatively regulate the BRCA1 protein expression in ER+ & PR+ & Triple negative breast cancer cells. However, there is no correlation found in MCF10A breast normal cells. From our correlation analysis of gene and protein expression we have observed that out of these three MBD proteins only *MBD2* gene regulate the *BRCA1* gene expression in ER+ & PR+ & Triple negative breast cancer cells, others only have regulation in ER+ & PR+ breast cancer cells. Wood et al. 2016 & Lin et al. 2003 has also reported that *MBD2* regulation in the gene expression and described its role in the development and differentiation of multiple cell lineages, including pluripotent stem cells and various cell types of the immune system, as well as in tumorigenesis. This could be due to the its structural sequence where MBD and TRD domain both overlap and binds to the promoter region strongly and regulate the gene expression as compare to the other MBD gene which has separate MBD and TRD domain in their sequence [8, 9].

To learn about the cellular effect of these genes further we have studied the colony formation in MCF-7 and MDA-MB-231 breast cancer cells. Here we found that colony formation was gradually reduced with increasing concentrations of resveratrol and it has significant inhibitory effect at 30µM. The same effect was reported by Zhao et al., 2018 that resveratrol inhibited colony formation via suppression of *N-cadherin, Snail, ERK1* and up-regulation of *E-cadherin* in renal cell carcinoma [49]. Similar outcomes were seen in migration assay to evaluate the metastatic role of MCF-7 and MDA-MB-231 cell line, which is significantly reduced migration at higher concentration after 24hr of resveratrol treatment; however there was no effect observed in MCF-10A control cell line. Xiong et al., 2016 also support this inhibitory effect of resveratrol on glioblastoma cells [50]. Further we have evaluated sphere-forming ability of resveratrol treated cells, indicating that sphere size of MCF-7, T-47D and MDA-MB231 cells increases at control cells and gradually decreases along with the increasing concentrations of resveratrol and significantly reduced at higher concentration (50µM) and there is no sphere formation observed in MCF-10A control cell line. Wu et al. 2019 reported that resveratrol arrest the G1 to S phase transition leading to cells proliferation inhibition that supports my observation in this study [51]. We have also checked the cytotoxic effect of resveratrol on the sphere of these cell lines and found that apoptotic cells no. increases along with the increasing concentrations of resveratrol and significant at higher concentration. We have also checked apoptosis in cells monolayer of breast cancer cells and found that increasing concentrations of resveratrol increases apoptotic cells and has significant at higher concentration but no effect was observed in MCF-10A control cell line. Similar effect of resveratrol reported in ovarian cancer cell and breast cancer 4T1 cells which strongly support our findings [51, 52].

**Fig. 7:**
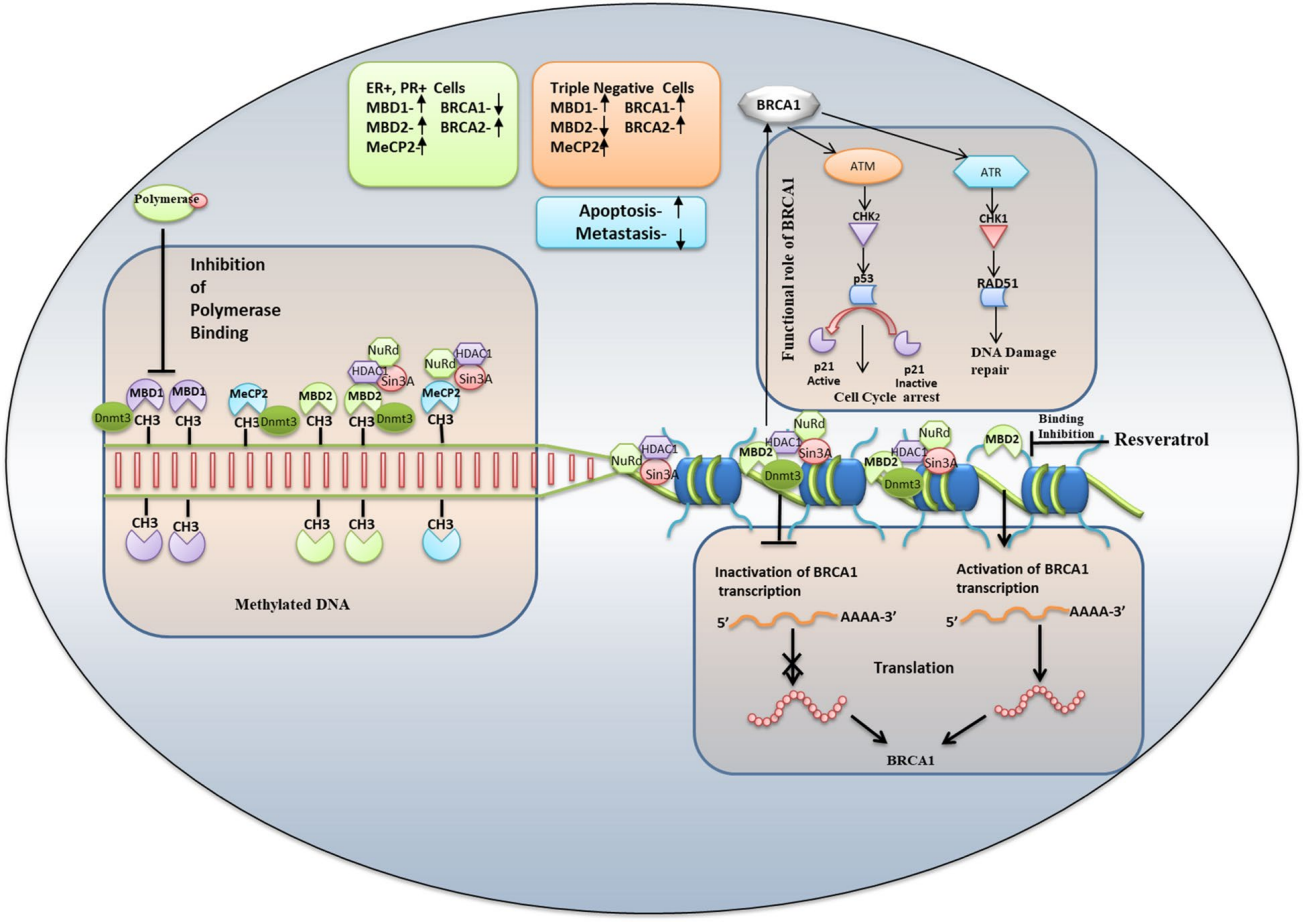
Conclusion of the study revealed that methyl-CpG binding protein binds to the *BRCA1* promoter and *MBD2* has regulatory role on *BRCA1* gene expression which can be modulated by resveratrol and induces apoptosis, inhibited metastasis in Breast cancer cells

In conclusion, Methyl CpG binding proteins bind to the promoter of *BRCA1* gene but not on *BRCA2, p16* gene. In ER+, PR+ breast cancer cells, *BRCA1* was down-regulated and *MBD1, MBD2, MeCP2, BRCA2,* & *p16* was up-regulated, but in Triple-negative breast cancer cells, *MBD2* was down-regulated and *MBD1, MeCP2, BRCA1, BRCA2,* & *p16* gene was elevated following treatment with resveratrol. Correlation analysis demonstrated that only *MBD2* has negative regulatory role on *BRCA1* gene expression as well as in protein expression in ER+, PR+ & Triple negative breast cancer cells; its *MBD2-BRCA1* axis indicates their significant role in the induction of apoptosis and reduction of sphere formation, colony formation, and metastasis in Breast cancer cells (Fig. 7). This study provides a detailed and intricate analysis of the interactions between DNA methylation, MBD proteins, and resveratrol in breast cancer cells. The identification of *MBD2* as a negative regulator of *BRCA1* expression, along with the functional consequences on cell behavior, adds depth to our understanding of epigenetic and pharmacological interventions in breast cancer. Our work contributes significantly to the growing body of knowledge in cancer epigenetics and highlights potential avenues for targeted therapies. The integrated approach, combining molecular, genetic, and functional analyses, strengthens the study’s impact and lays the groundwork for future research in this field.

This study recognizes the need for further research, especially in vivo and clinical settings, to validate the observed results. Exploring mutations in MBD1, MBD2, and MeCP2 proteins and their implications on breast cancer progression is identified as a crucial area for future investigation.

### Supplementary Information

**Supplementary Material 1.**

## Data Availability

The data that support the findings of this study are available in the supplementary files.
