# Supplementary material for: Methyl CpG binding protein MBD2 has a regulatory role on the BRCA1 gene expression and its modulation by resveratrol in ER+, PR+ & triple-negative breast cancer cells
Source: BMC Cancer. 2024 May 6;24:566. doi: 10.1186/s12885-024-12274-x (PMC11071212; doi:10.1186/s12885-024-12274-x)
Supplement: Supplementary file 1 — Supplementary Material 1. [file 12885_2024_12274_MOESM1_ESM.zip › Supplementary file-gel image 1.pdf]

Supplementary Figure 2. A-I) Original unprocessed image of promoter binding of MBD1, MBD2 & MeCP2 proteins on BRCA1, BRCA2 & p16 genes were analyzed by EMSA assay on 8% PAGE gel and transferred in nylon membrane then exposed in X-ray film. Shifting of bands was observed by using protein specific primary antibody arrow showing in between image indicate the bands.

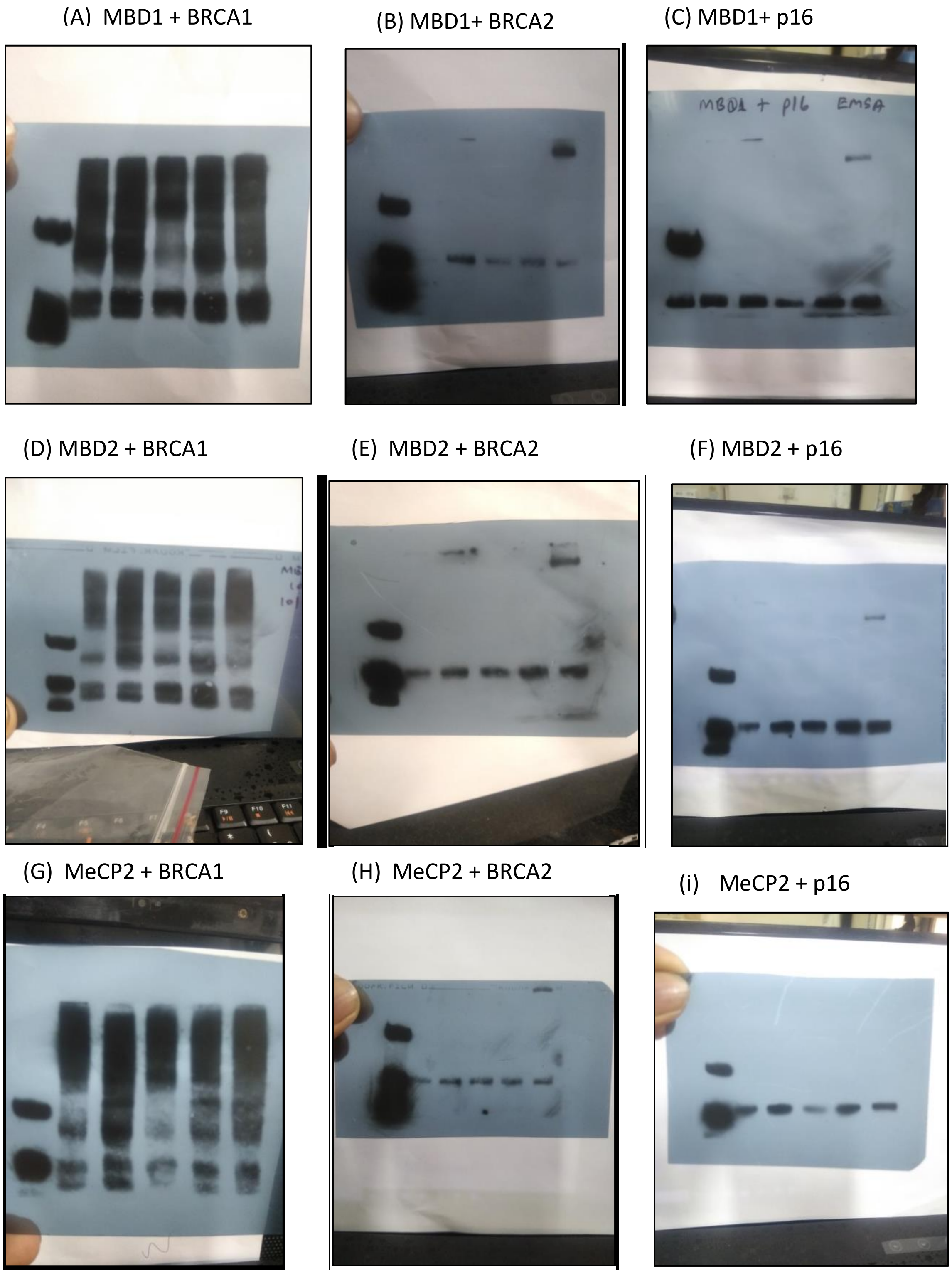

Supplementary Figure 2. J) Original unprocessed image of chromosome immune precipitation of BRCA1, BRCA2 & p16 gene was done by ChIP assay using MBD1, MBD2 & MeCP2 primary antibody and amplification of these genes were done by PCR and run on agarose gel and bands were observed.

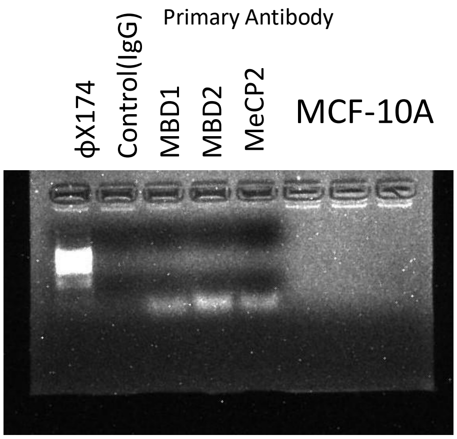

Figure: ChIP Assay of BRCA1 (86bp) was done by using MBD1, MBD2 & MeCP2 primary antibody in MCF10A, MCF-7, T-47D & MDA-MB-231 breast normal and cancer cell line respectively and amplification of these genes were done by PCR and run on agarose gel and bands were observed.

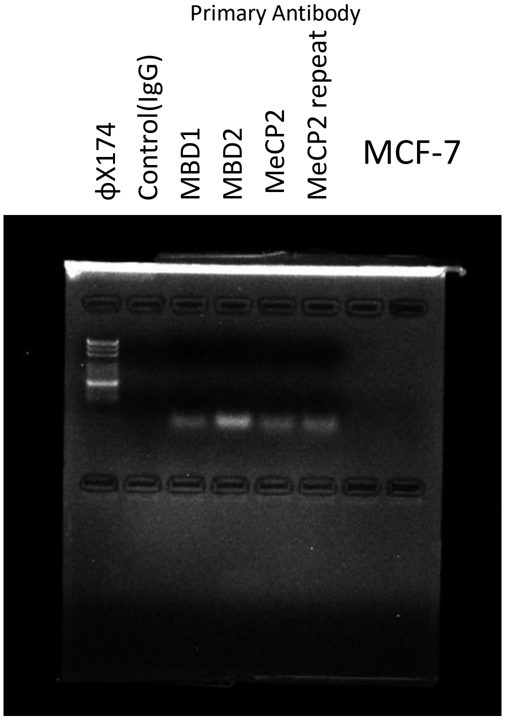

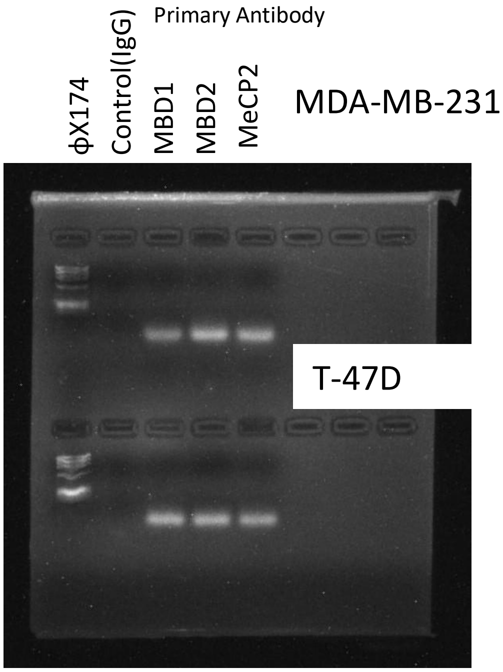

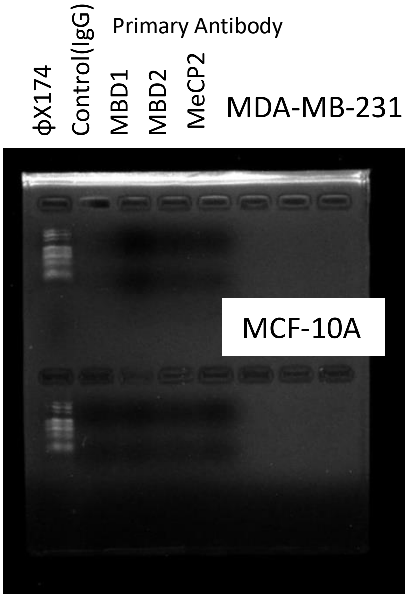

Figure: ChIP Assay of BRCA2 (60bp) was done by using MBD1, MBD2 & MeCP2 primary antibody in MCF10A, MCF-7, T-47D & MDA-MB-231 breast normal and cancer cell line respectively and amplification of these genes were done by PCR and run on agarose gel and bands were observed.

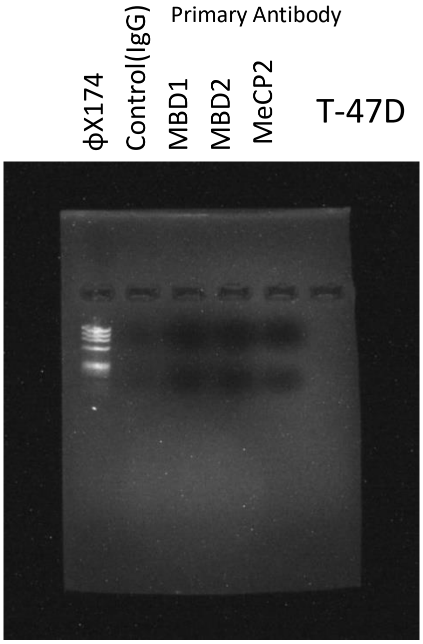

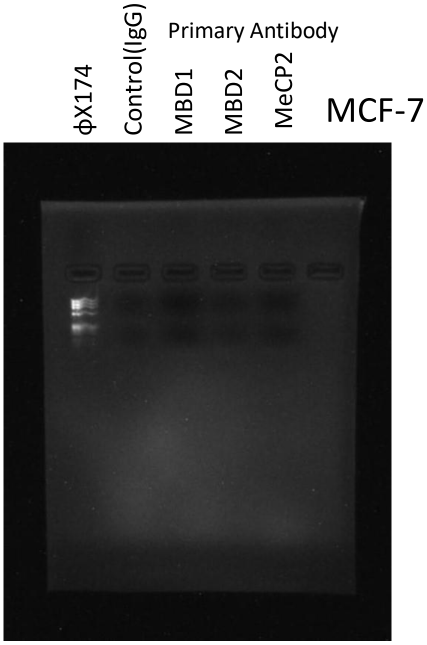

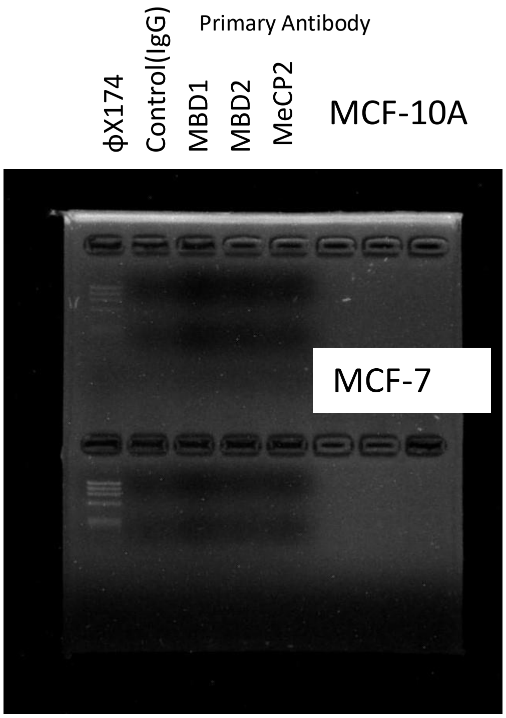

Figure: ChIP Assay of p16 (151bp) was done by using MBD1, MBD2 & MeCP2 primary antibody in MCF10A, MCF-7, T-47D & MDA-MB-231 breast normal and cancer cell line respectively and amplification of these genes were done by PCR and run on agarose gel and bands were observed.

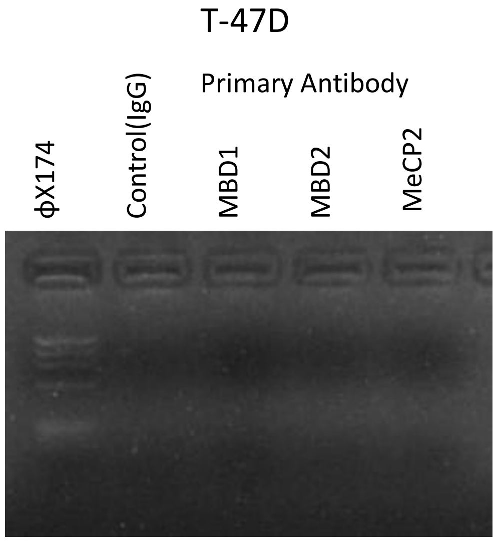

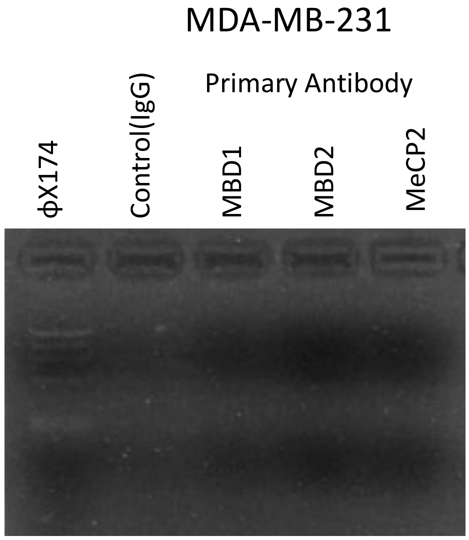

Supplementary Figure 2. K) Original unprocessed image of Methylation immune precipitation assay of BRCA1, BRCA2 & p16 genes were done by MeIP assay using 5mC methylation specific primary antibody in MCF10A, MCF-7, T-47D & MDA-MB-231 breast normal and cancer cell line respectively and amplification of these genes were done by PCR and run on 10% agarose gel to observed bands.

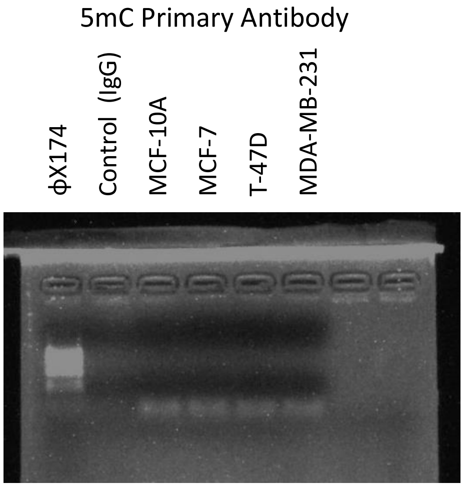

Methylation immune precipitation assay of BRCA1 using 5mC methylation specific primary antibody and amplification of these genes were done by PCR run on 10% agarose gel to observed bands.

BRCA1 (75bp)

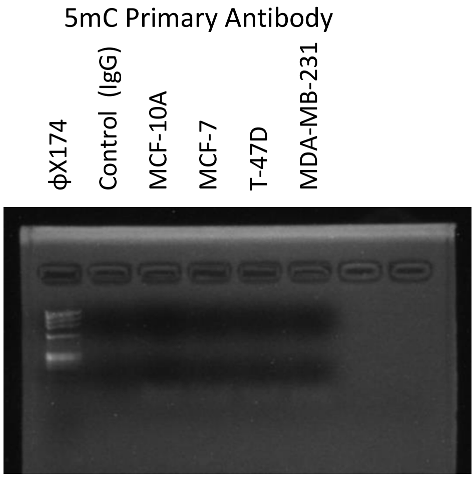

Methylation immune precipitation assay of BRCA2 using 5mC methylation specific primary antibody and amplification of these genes were done by PCR run on 10% agarose gel to observed bands.

BRCA2 (62bp)

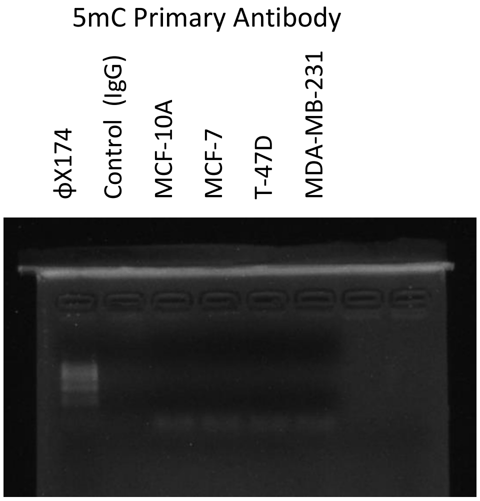

Methylation immune precipitation assay of p16 using 5mC methylation specific primary antibody and amplification of these genes were done by PCR run on 10% agarose gel to observed bands.

p16 (150bp)

Table.1:- List of probe and primer sequence of BRCA1, BRCA2 & p16 gene used in EMSA for binding assay and ChIP & MeIP assay for amplification of these genes and RT primer for the real time expression of MBD1, MBD2, MeCP2, BRCA1, BRCA2 & p16 gene.
