# Supplementary material for: Methyl CpG binding protein MBD2 has a regulatory role on the BRCA1 gene expression and its modulation by resveratrol in ER+, PR+ & triple-negative breast cancer cells
Source: BMC Cancer. 2024 May 6;24:566. doi: 10.1186/s12885-024-12274-x (PMC11071212; doi:10.1186/s12885-024-12274-x)
Supplement: Supplementary file 1 — Supplementary Material 1. [file 12885_2024_12274_MOESM1_ESM.zip › Supplementary file-Western blotting 1.pdf]

Supplementary Figure 4: Protein expression of *MBD1, MBD2, MeCP2, BRCA1, BRCA2 & p16* genes were done by western blotting in resveratrol treated MDA-MB-231, MCF-7, T-47D & MCF-10A breast normal and cancer cell lines. Bands were transferred to PVDF membrane and exposed to X-ray film and processed images were place in the results. Densitometry analysis was done by MyImage analysis software (thermo Scientific) to quantify the bands intensity and normalized with housekeeping β-actin gene.

Figure (A): Protein expression of *MBD1, MBD2, MeCP2, BRCA1, BRCA2 & p16* genes were done by western blotting in resveratrol treated MCF-10A breast normal cell line Bands were transferred to PVDF membrane and exposed to X-ray film.

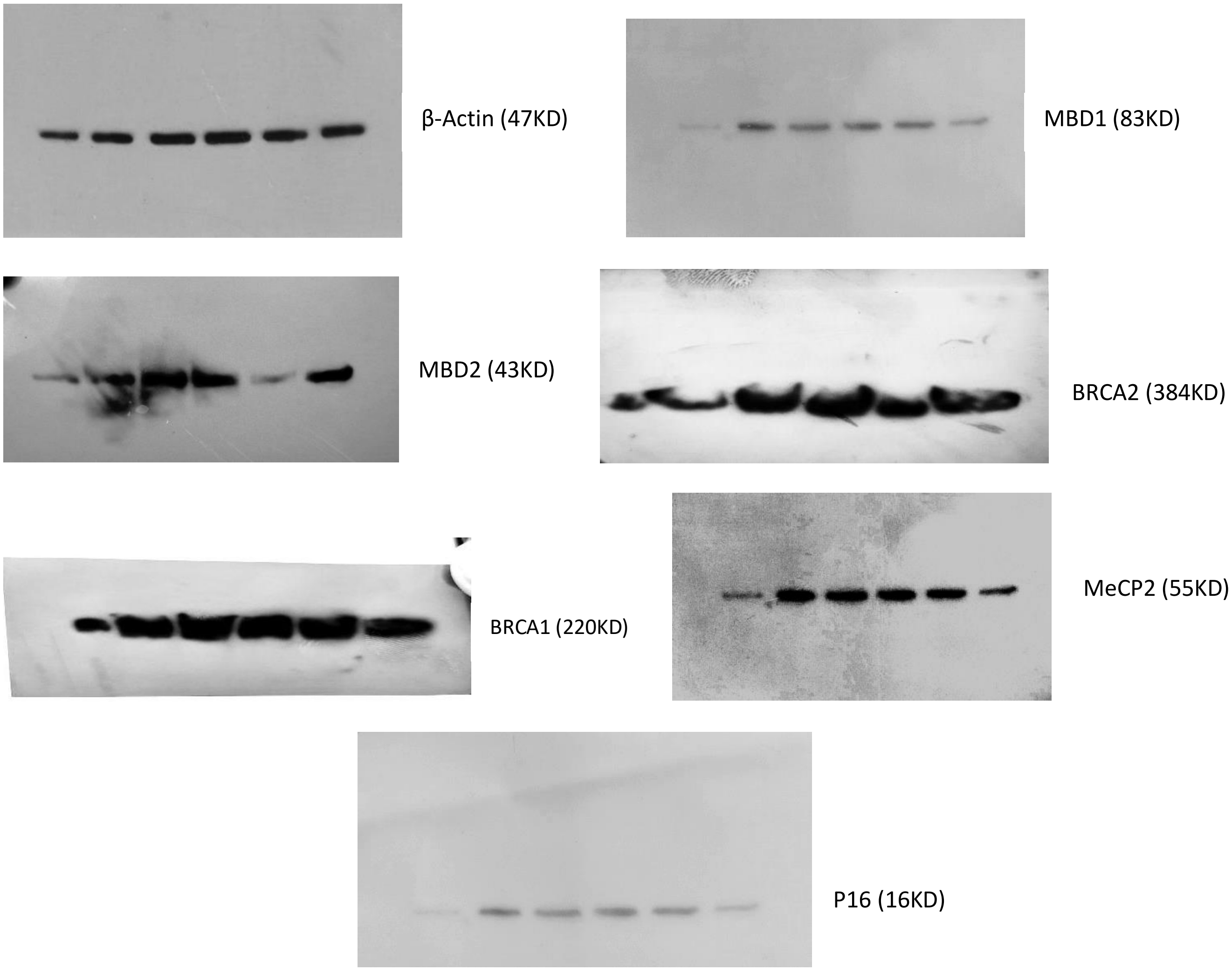

Figure (B): Protein expression of *MBD1*, *MBD2*, *MeCP2*, *BRCA1*, *BRCA2 & p16* genes were done by western blotting in resveratrol treated MCF-7 breast cancer cell line Bands were transferred to PVDF membrane and exposed to X-ray film.

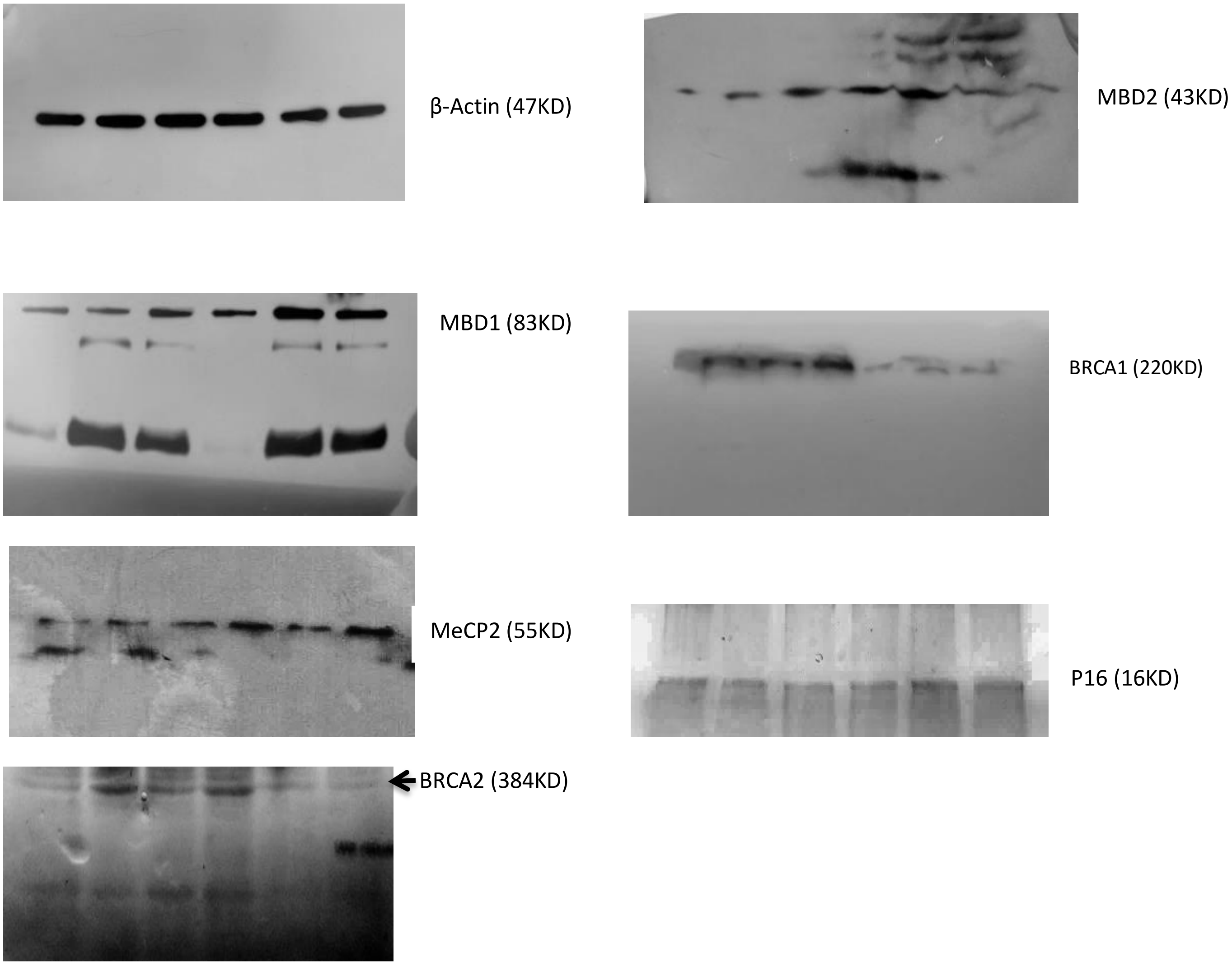

Figure (C): Protein expression of *MBD1, MBD2, MeCP2, BRCA1, BRCA2 & p16* genes were done by western blotting in resveratrol treated MDA-MB-231 breast cancer cell line Bands were transferred to PVDF membrane and exposed to X-ray film.

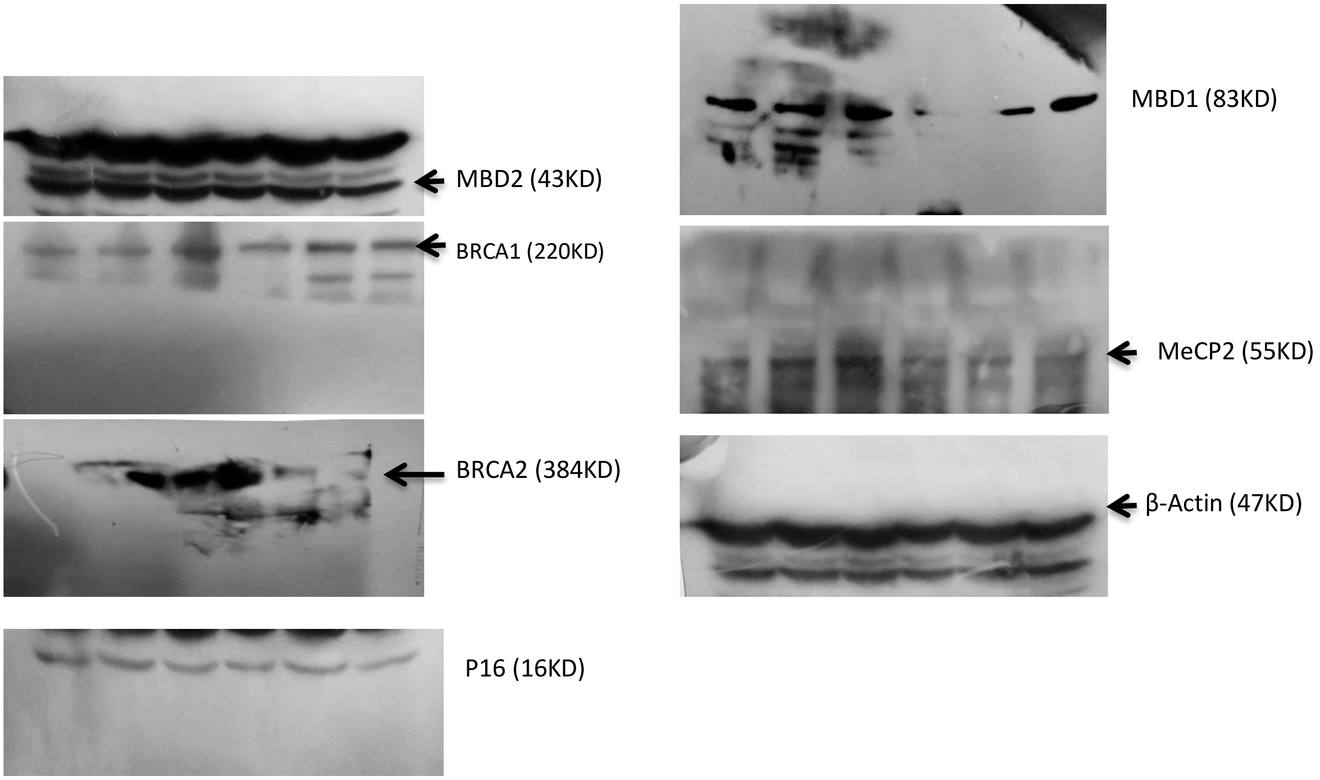

Figure (D): Protein expression of *MBD1, MBD2, MeCP2, BRCA1, BRCA2 & p16* genes were done by western blotting in resveratrol treated T-47D breast cancer cell line Bands were transferred to PVDF membrane and exposed to X-ray film.

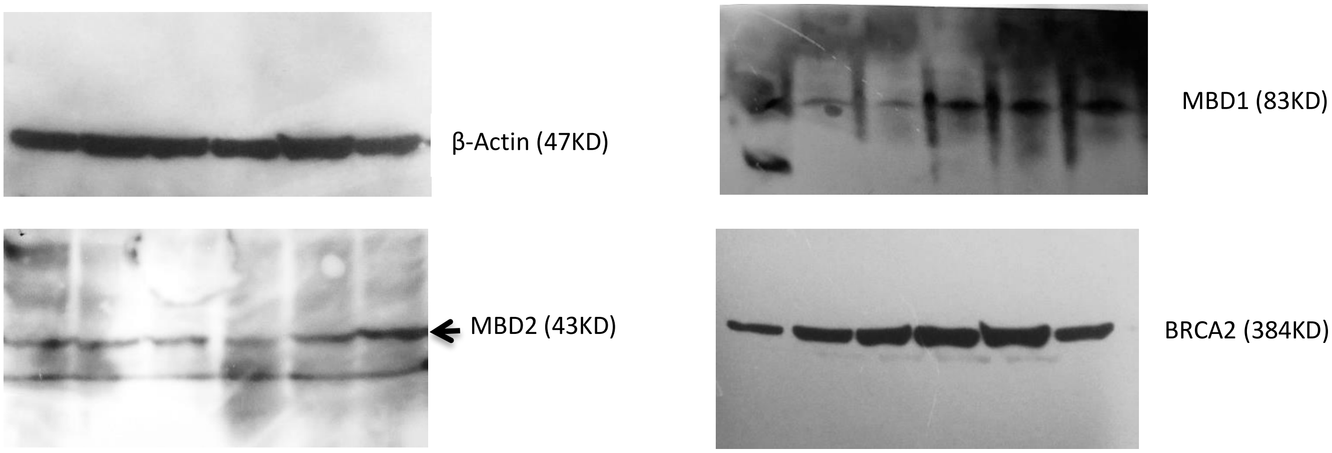

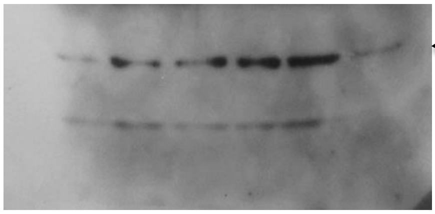

MeCP2 (55KD)

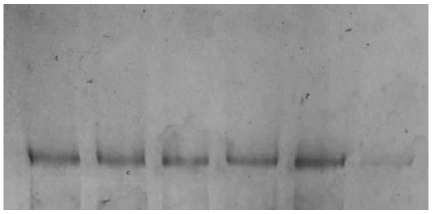

P16 (16KD)

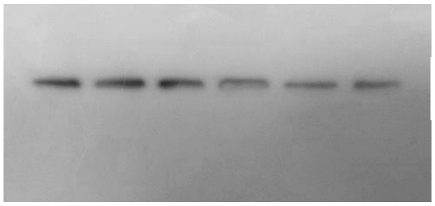

BRCA1 (220KD)
