# Supplementary material for: Methyl CpG binding protein MBD2 has a regulatory role on the BRCA1 gene expression and its modulation by resveratrol in ER+, PR+ & triple-negative breast cancer cells
Source: BMC Cancer. 2024 May 6;24:566. doi: 10.1186/s12885-024-12274-x (PMC11071212; doi:10.1186/s12885-024-12274-x)
Supplement: Supplementary file 1 — Supplementary Material 1. [file 12885_2024_12274_MOESM1_ESM.zip › Supplementry file. Western blotting Image-1.pdf]

# MCF-10A (Fig-4)

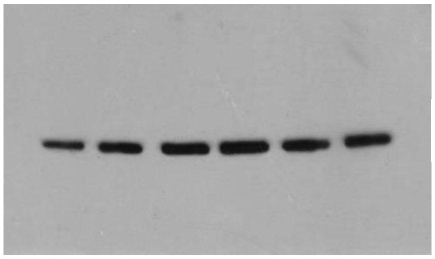

β-Actin (47KD)

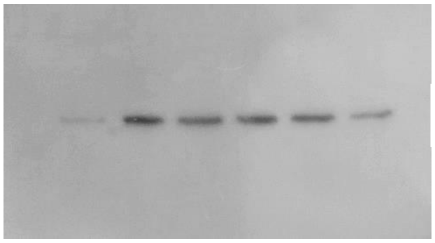

MBD1 (83KD)

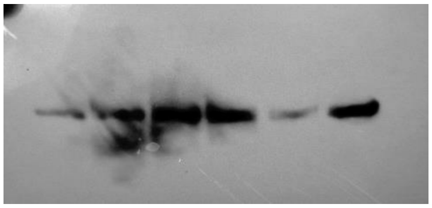

MBD2 (43KD)

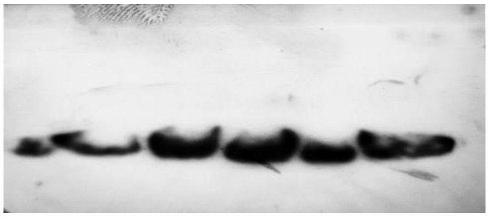

BRCA2 (384KD)

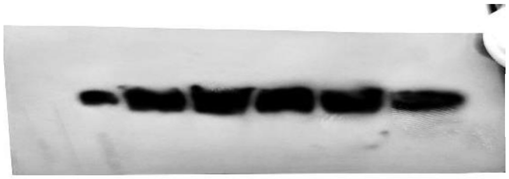

BRCA1 (220KD)

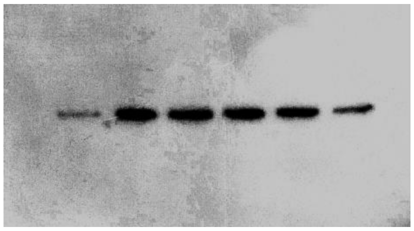

MeCP2 (55KD)

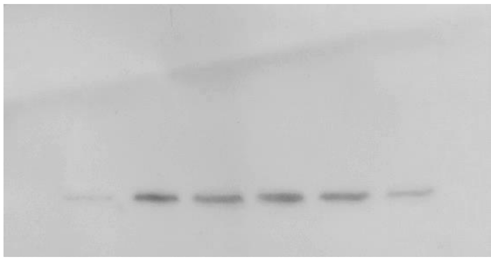

P16 (16KD)

# MCF-7(Fig-4)

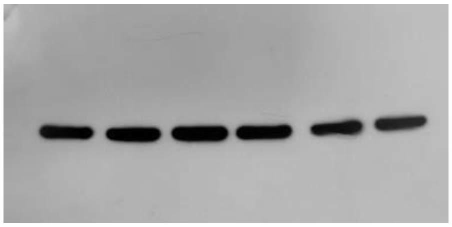

β-Actin (47KD)

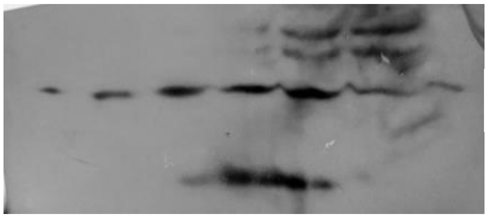

MBD2 (43KD)

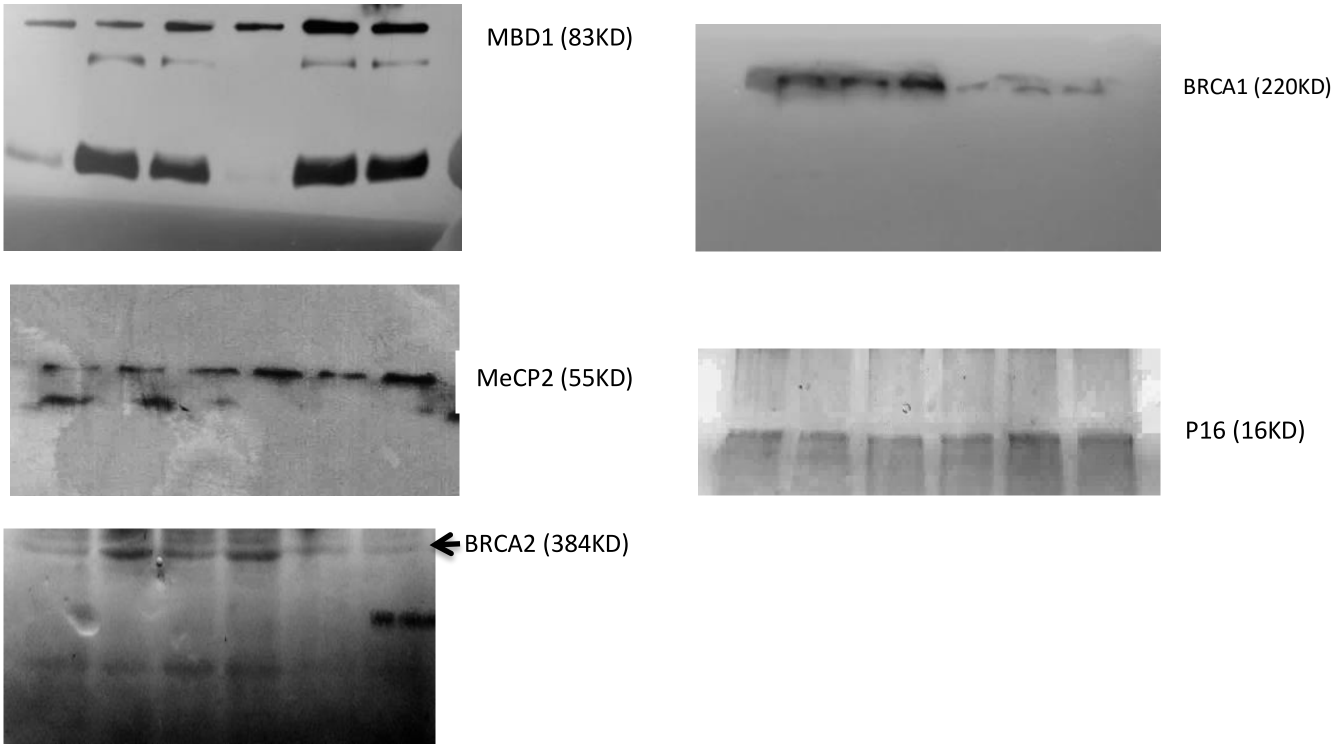


**MDA-MB-231(Fig-4)**

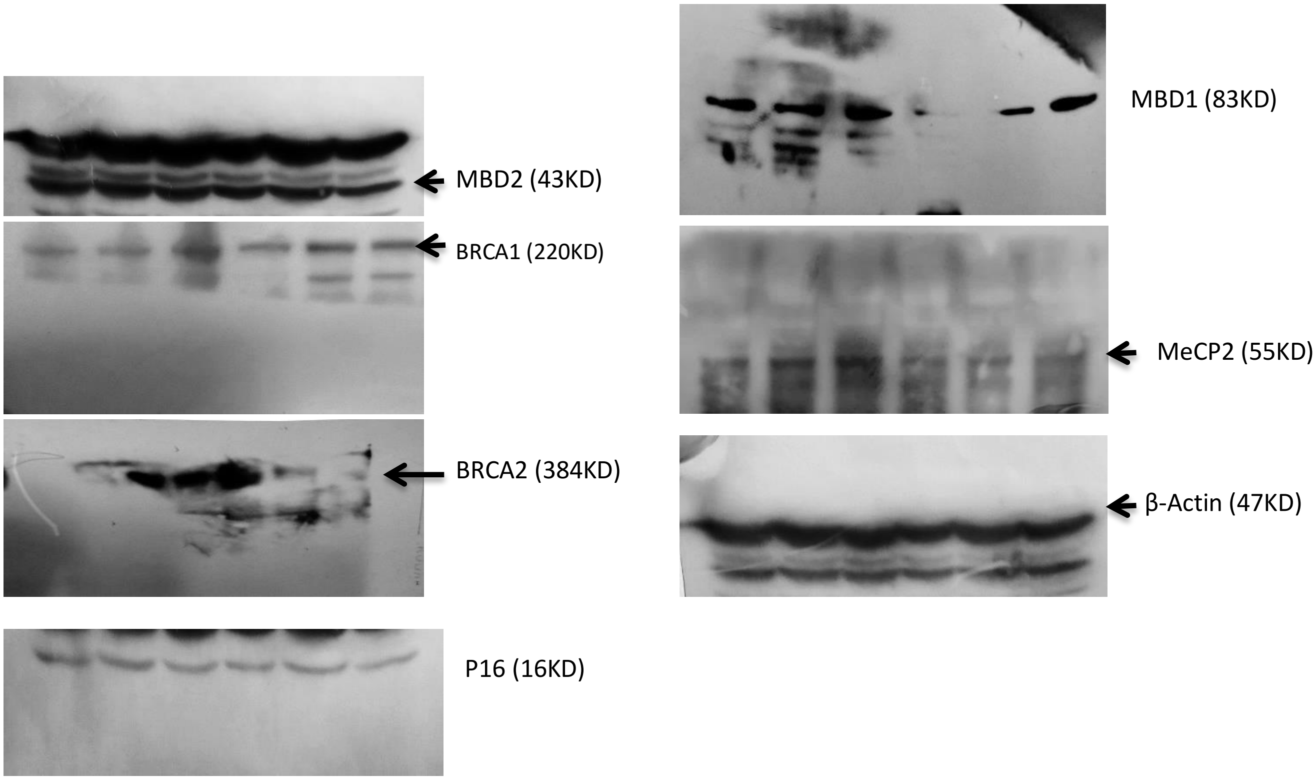

## T-47D (Fig-4)

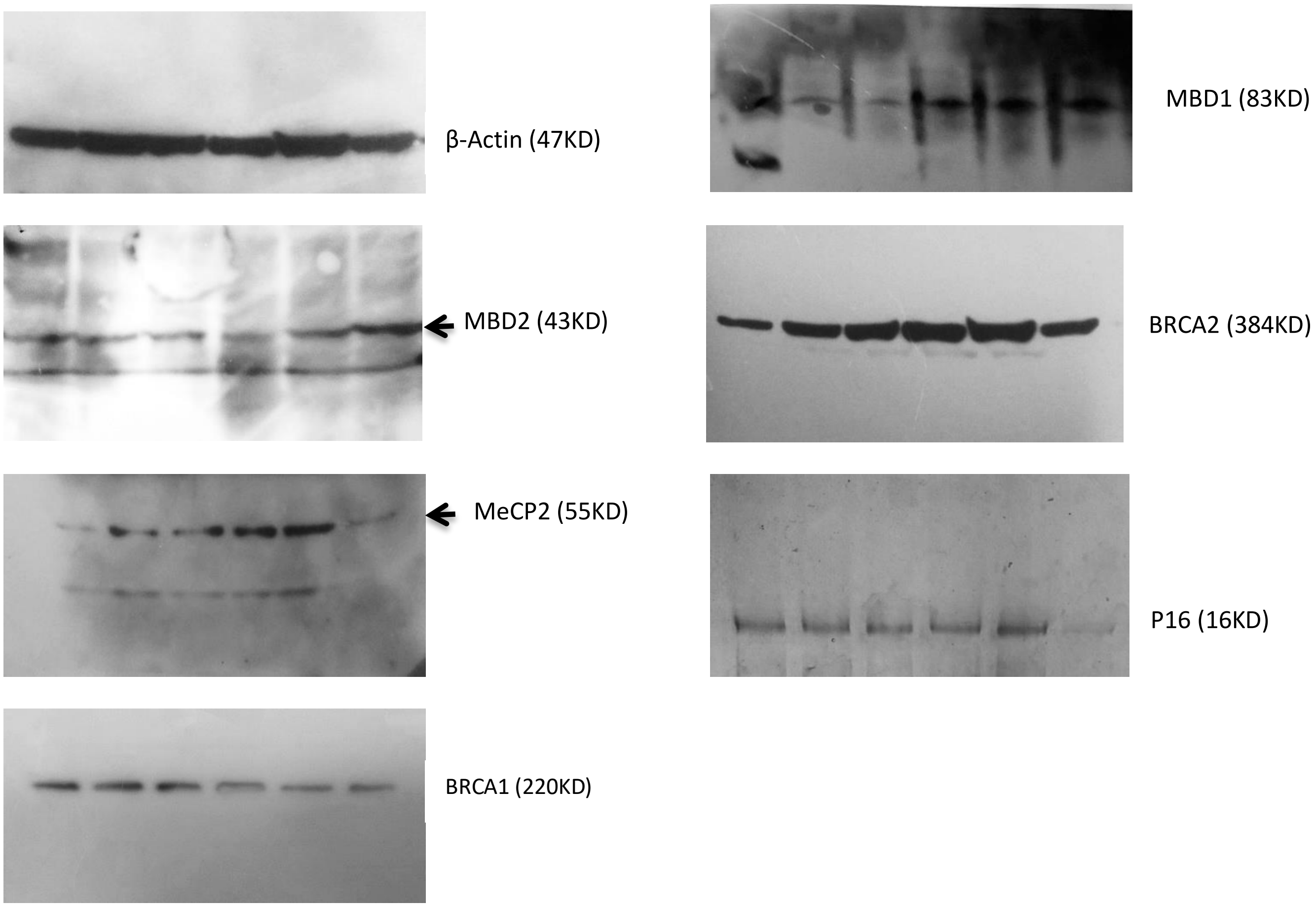
β-Actin (47KD)
MBD1 (83KD)
MBD2 (43KD)
BRCA2 (384KD)
MeCP2 (55KD)
P16 (16KD)
BRCA1 (220KD)
