# Supplementary figures and images for: Methyl CpG binding protein MBD2 has a regulatory role on the BRCA1 gene expression and its modulation by resveratrol in ER+, PR+ & triple-negative breast cancer cells
Source: BMC Cancer. 2024 May 6;24:566. doi: 10.1186/s12885-024-12274-x (PMC11071212; doi:10.1186/s12885-024-12274-x)

ChIP Assay of BRCA1 (86bp)

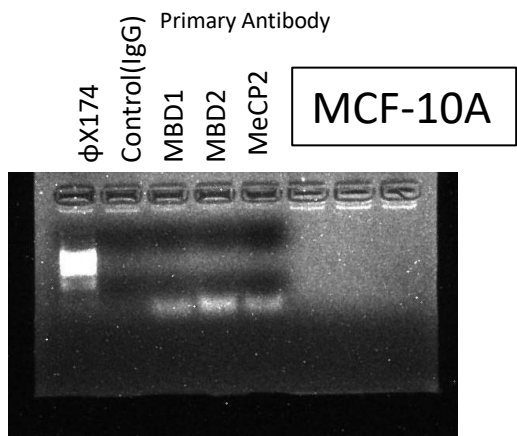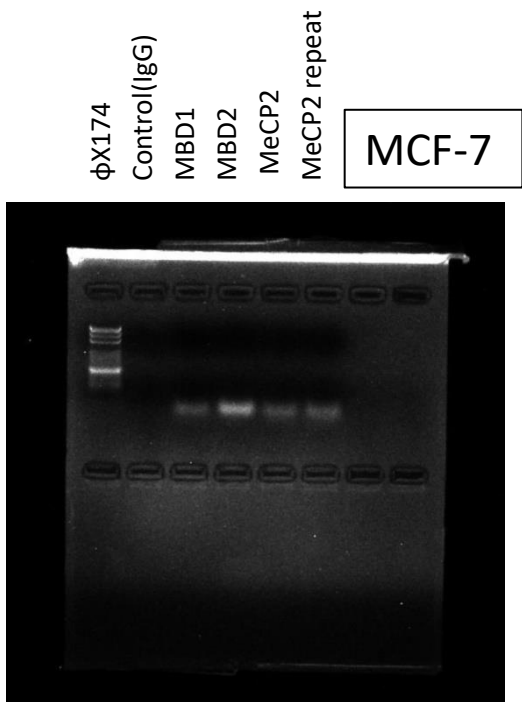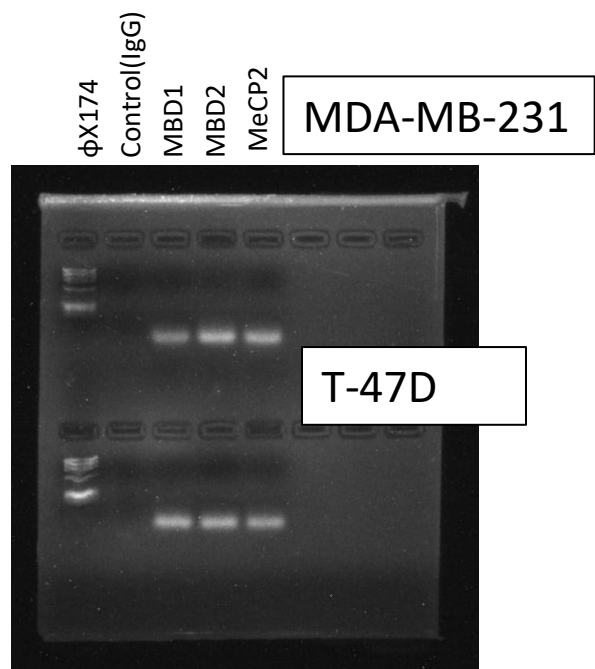

ChIP Assay of BRCA2 (60bp)

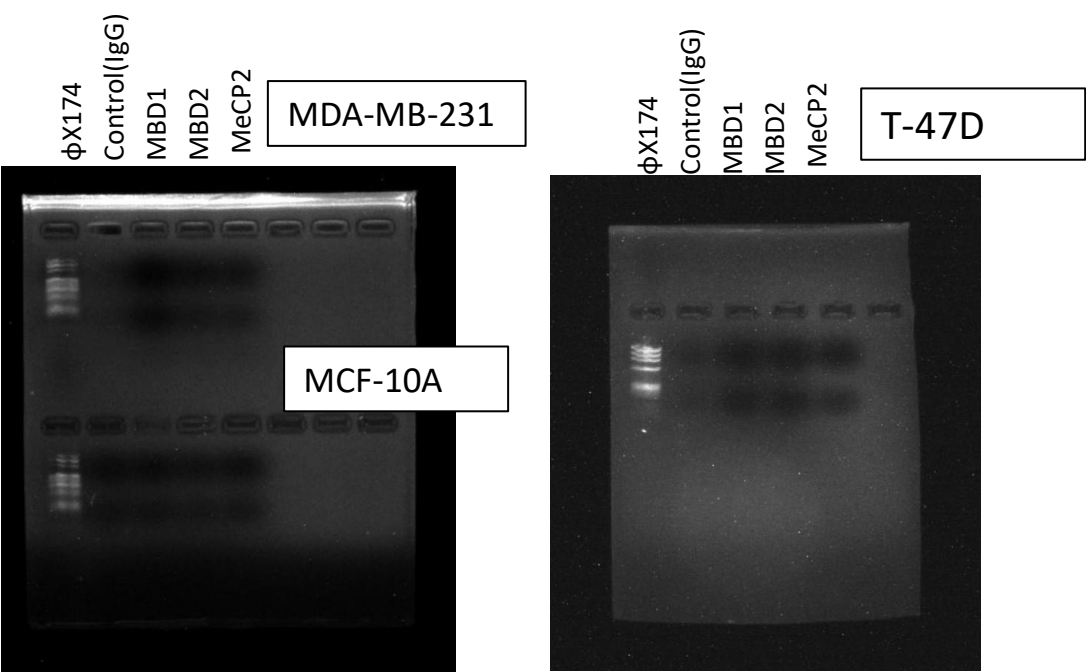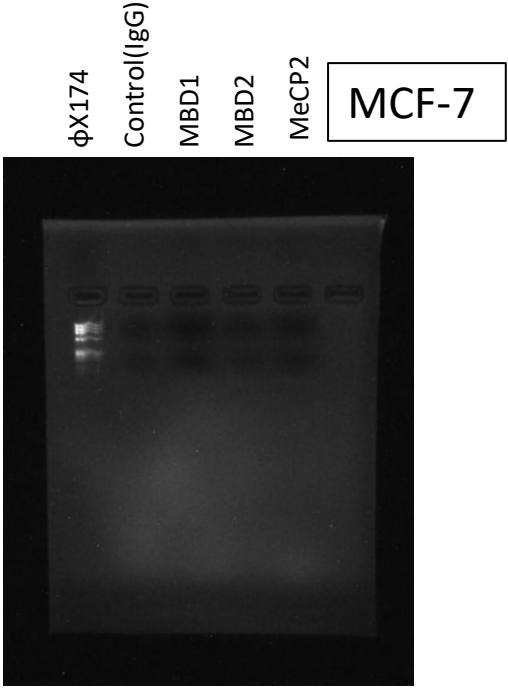

## ChIP Assay of p16 (151bp)

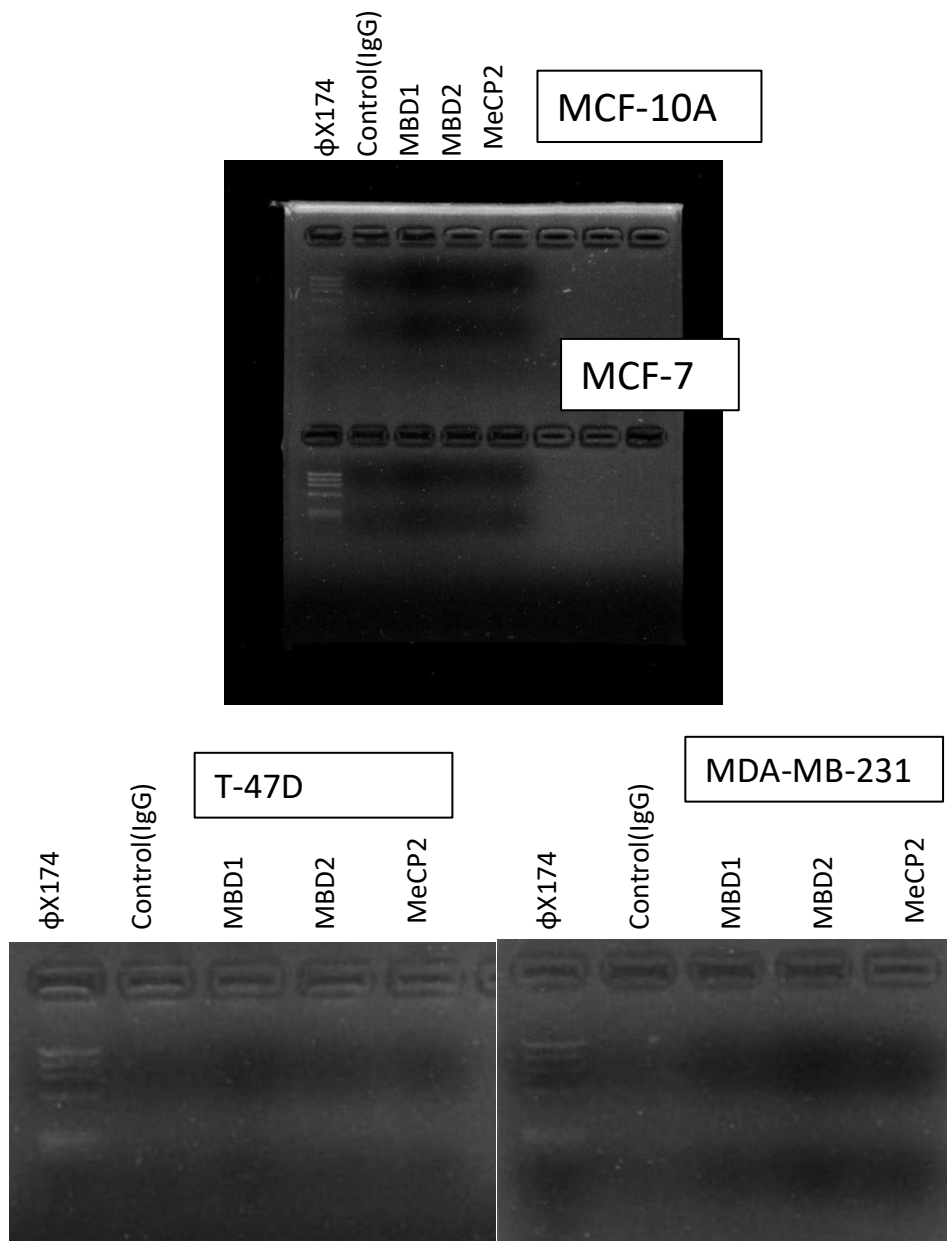

## MeIP Assay

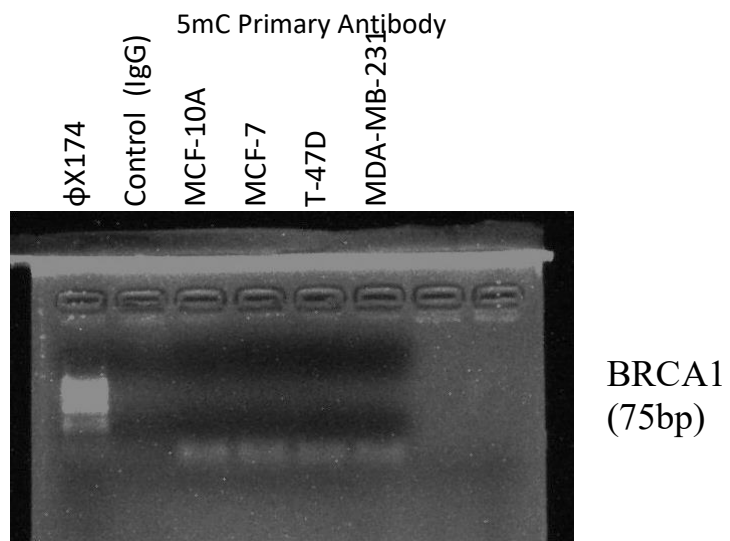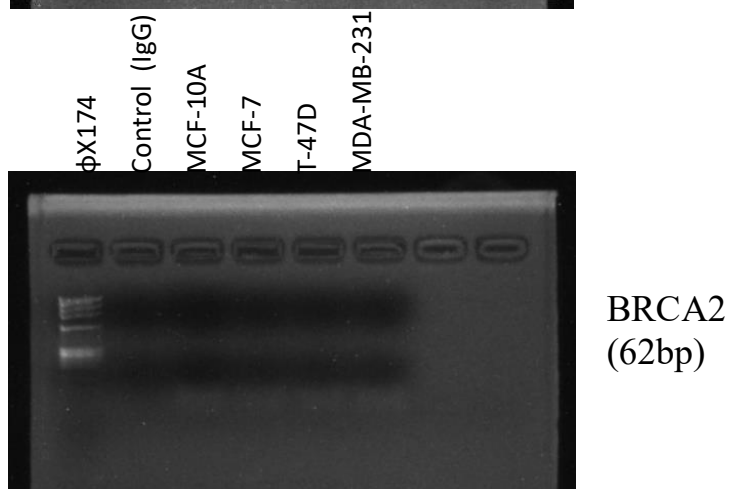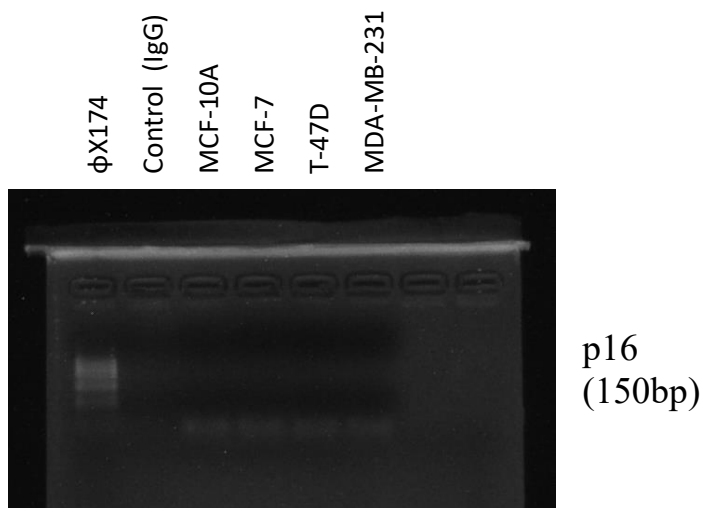

Supplement: Supplementary file 1 — Supplementary Material 1. [file 12885_2024_12274_MOESM1_ESM.zip › Supplimentory file-gel image.pdf]
